# Risk of antimicrobial-associated organ injury among the older adults: a systematic review and meta-analysis

**DOI:** 10.1186/s12877-021-02512-3

**Published:** 2021-11-01

**Authors:** Tichawona Chinzowu, Sandipan Roy, Prasad S. Nishtala

**Affiliations:** 1 Department of Pharmacy and Pharmacology & Centre for Therapeutic Innovation, University of Bath, Bath, BA2 7AY UK; 2 Department of Mathematical Science, University of Bath, Bath, BA2 7AY UK

**Keywords:** Systematic review, meta-analysis, Older adults, Antimicrobial, Organ injury

## Abstract

**Background:**

Older adults (aged 65 years and above) constitute the fastest growing population cohort in the western world. There is increasing evidence that the burden of infections disproportionately affects older adults, and hence this vulnerable population is frequently exposed to antimicrobials. There is currently no systematic review summarising the evidence for organ injury risk among older adults following antimicrobial exposure. This systematic review and meta-analysis examined the relationship between antimicrobial exposure and organ injury in older adults.

**Methodology:**

We searched for original research articles in PubMed, Embase.com, Web of Science core collection, Web of Science BIOSIS citation index, Scopus, Cochrane Central Register of Controlled Trials, ProQuest, and PsycINFO databases, using key words in titles and abstracts, and using MeSH terms. We searched for all available articles up to 31 May 2021. After removing duplicates, articles were screened for inclusion into or exclusion from the study by two reviewers. The Newcastle-Ottawa scale was used to assess the risk of bias for cohort and case-control studies. We explored the heterogeneity of the included studies using the Q test and I^2^ test and the publication bias using the funnel plot and Egger’s test. The meta-analyses were performed using the OpenMetaAnalyst software.

**Results:**

The overall absolute risks of acute kidney injury among older adults prescribed aminoglycosides, glycopeptides, and macrolides were 15.1% (95% CI: 12.8–17.3), 19.1% (95% CI: 15.4–22.7), and 0.3% (95% CI: 0.3–0.3), respectively. Only 3 studies reported antimicrobial associated drug-induced liver injury. Studies reporting on the association of organ injury and antimicrobial exposure by age or duration of treatment were too few to meta-analyse. The funnel plot and Egger’s tests did not indicate evidence of publication bias.

**Conclusion:**

Older adults have a significantly higher risk of sustaining acute kidney injury when compared to the general adult population. Older adults prescribed aminoglycosides have a similar risk of acute kidney injury to the general adult population.

**Supplementary Information:**

The online version contains supplementary material available at 10.1186/s12877-021-02512-3.

## Background

Older adults aged 65 years and above comprise the fastest and largest expanding population age group in the developed world [1]. They are prone to infectious diseases such as pneumonia, skin and soft tissue infections (SSTI), urinary tract infections (UTI) and septicaemia when compared to younger people [1]. It is estimated that the older adults comprise 48.7% of individuals admitted to hospital intensive care units for these infections [2], resulting in their increased length of hospital stay and exposure to antimicrobials. Giarratano et al. [3] highlighted several predisposing factors that make older adults more susceptible to antimicrobial adverse events. These include physiological changes, higher comorbidities, drug-drug interactions, drug delivery routes used, and length of time they are in contact or exposed to the antimicrobial agents. In one large prospective cohort study, antimicrobial related adverse events accounted for 19.3% of all drug-related adverse events seen at the emergency department [2]. Several antimicrobial-associated adverse events become apparent years after the drug has been approved. The adverse events reported in clinical trials differ considerably from post-marketing surveillance [4]. Since most clinical trials exclude older adults, the true nature and incidence of antimicrobial related adverse events in this population are unknown. In their review, Giarratano et al. [3] concluded that there is a general lack of epidemiological studies on antimicrobials used among the older adults, yet this is essential in informing healthcare providers to achieve optimal safety and effectiveness when providing antimicrobial pharmacotherapy to the older adults.

This paper describes a systematic review and meta-analysis carried out to investigate the risk of antimicrobial-associated kidney, liver, or tissue injury among older adults. The main outcome was quantifying the association of organ injury among older adults following exposure to antimicrobials.

## Methods

This systematic review was conducted after the study protocol was registered with PROSPERO (CRD4202015262).

### Search strategy and selection criteria

For this systematic review and meta-analysis, we searched for original research articles for observational studies describing kidney, liver or tissue injury associated with exposure to antimicrobials among older adults (65 years or above). We restricted our searches to the English language only for all available articles up to 31 May 2021.

Searches were conducted in PubMed, Embase.com, Web of Science core collection, Web of Science BIOSIS citation index, Scopus, Cochrane Central Register of Controlled Trials, ProQuest, and PsycINFO databases, using key words in titles and abstracts, and using MeSH terms. The full search criteria for all the databases are in additional file 1, document 1. After removing duplicates, TC carried out the title and abstract screening on all the articles for preliminary inclusion into the systematic review. PN and SR independently carried out title and abstract screening of 50% of the articles each for preliminary inclusion into the systematic review. Among the preliminary included articles, TC and PN carried out full article review of the first 50% of the articles, and TC and SR reviewed the other half. Where there were disagreements between reviewers in both the preliminary review and full article review, the opinion of the third reviewer was sought.

### Inclusion and exclusion criteria

Studies were included in the systematic review and meta-analysis if they reported the absolute risk of antimicrobial-associated kidney, liver, or tissue injury among older adults. Studies were also included if they reported a subgroup analysis of participants who met the inclusion criteria, thus 65 years or older. Studies were excluded if they included adults below 65 years of age without relevant subgroup analysis for those 65 years or older; the outcome of interest was not kidney, liver, or tissue injury; the exposure was not an antimicrobial; and the study design was not an original observational study.

### Risk of bias assessment

The Newcastle-Ottawa scale [5] was used to assess the risk of bias for each observational study (see additional file 4, document 1). Each study that scored 6 or more points on the Newcastle-Ottawa scale was considered to have a low risk of bias provided it scored at least 3 points on the selection domain, one or more on comparability, and at least 2 points on the outcome domain. All studies considered to be of low risk to bias were further considered for meta-analysis.

### Data analysis

Data extraction was completed by TC and PN independently, using a pre-piloted excel form. Data for the following variables were extracted: author details, country of study, study design, study setting, data source, any condition treated with antimicrobial, inclusion and exclusion criteria, outcome measurement and ascertainment, controlled confounders, sample size, exposure antibiotic, and absolute risk or odds ratio of organ injury among the participants. The full data extraction table is available as additional file 2, document 1.

Heterogeneity among the studies included for meta-analysis was assessed using the Cochrane Q statistic [6] at 5% significance level and quantified using Higgins and Thompson’s I^2^ statistic [6]. An I^2^ value of more than 50% was considered to reflect substantial heterogeneity, and therefore, sensitivity analysis to investigate the possible source of heterogeneity was done. For the meta-analysis results, publication bias was assessed using a funnel plot [7] and Egger’s regression test [8]. Regardless of the heterogeneity, the random-effects model, using the DerSimonian and Laird method [9], was used to meta-analyse the studies. The meta-analyses were performed using the OpenMetaAnalyst software [10].

## Results

### Study identification

**Fig. 1:**
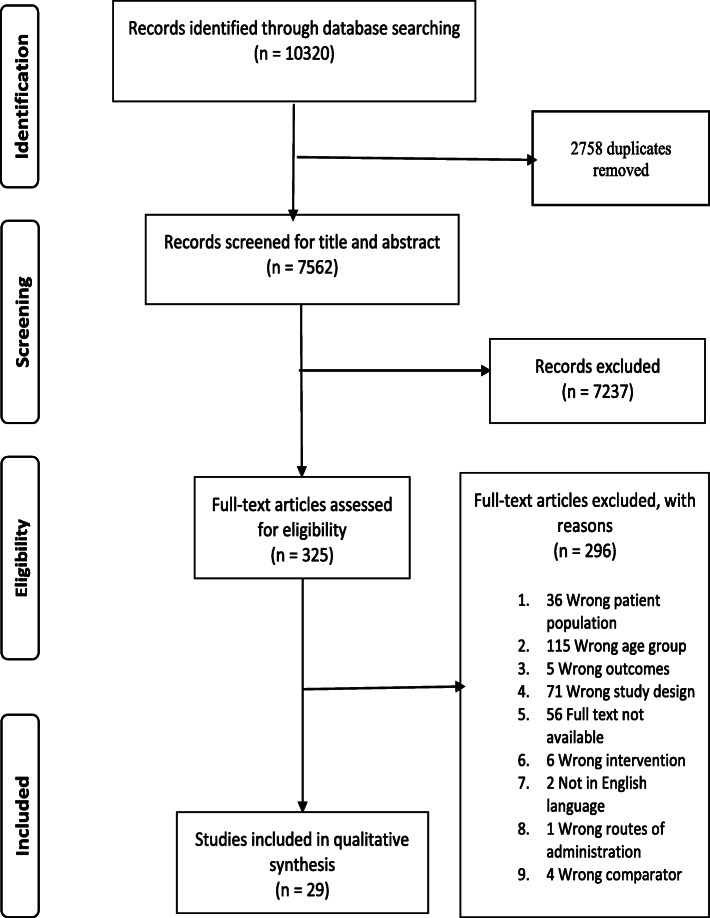
Flow chart of the literature search strategy to identify studies on antimicrobial exposure and organ injury risk

Following database searching, 10,320 studies were identified from the following databases: PubMed (1164), Embase.com (4880), Web of Science core collection (250), Web of Science BIOSIS citation index (301), Scopus (2901), Cochrane Central Register of Controlled Trials (817), ProQuest (6), and PsycINFO (1), of which 2758 were duplicates. Of the remaining 7562 studies, 7237 studies were excluded following title and abstract screening. A further 296 studies were excluded after the full-text screening, leaving 29 studies for inclusion into the the systematic review [11–39]. The full selection process is summarised in Fig. 1 below. Six of the included studies had data for two or more antimicrobials [16–18, 24, 27, 37], as shown in Table 1 below.

**Table 1:** Main characteristics of the included studies on antimicrobials and organ injury

Author, Year	Country	Method	Study Setting	Tool used to ascertain the outcome	Confounders	Sample size	Antibiotic class	Outcome	NOS Risk of bias
Aggarwal, 2018 [31]	India	Prospective cohort	Hospital setting	Laboratory values	Age, gender, BMI, APACHE 2, DM, antibiotic dose	26	Polymyxin	AKI	Low
Ahmed, 2016 [38]	UK	Retrospective cohort	Hospital setting	AKIN	age, gender, surgeon grade, type of anaesthesia	1500	Aminoglycoside	AKI	Low
Ahmed, 2019 [37]	UK	Retrospective cohort	Primary care	ICD-10 codes	age, sex, coronary heart disease, renal disease, liver disease, respiratory disease, type 2 diabetes mellitus, heart failure	28,800	Cephalosporin	AKI	Low
Ahmed, 2019 [37]	UK	Retrospective cohort	Primary care	ICD-10 codes	age, sex, coronary heart disease, renal disease, liver disease, respiratory disease, type 2 diabetes mellitus, heart failure	21,048	Penicillin	AKI	Low
Ahmed, 2019 [37]	UK	Retrospective cohort	Primary care	ICD-10 codes	age, sex, coronary heart disease, renal disease, liver disease, respiratory disease, type 2 diabetes mellitus, heart failure	14,868	Quinolone	AKI	Low
Ahmed, 2018 [11]	UK	Retrospective cohort	Primary care	ICD-10 codes	age, diabetes, dementia, coronary heart disease, stroke, cancer, heart failure, polypharmacy	29,936	Trimethoprim	AKI	Low
Baciewicz, 2003 [12]	USA	Prospective cohort	Hospital setting	Laboratory values	age, sex, nephrotoxic agents, diabetes mellitus, coronary artery disease, hypertension and	86	Aminoglycoside	AKI	Low
Baghaei, 2010 [33]	Iran	Prospective cohort	Hospital setting	Laboratory values	Age, sex, concomitant hepatotoxic drugs	295	antituberculosis	DILI	Low
Baik, 2020 [36]	USA	Prospective cohort	Population based	ICD-9 codes	Concurrent antibiotics, gender, race, rural residency, income status	685,018	Fluoroquinolone	Tendon rupture	Low
Bright-Thomas, 2016 [13]	UK	Retrospective cohort	Primary care	Laboratory values	age, sex, ethnic origin	200	Antituberculosis drugs	DILI	Low
Carreno, 2013 [14]	USA	Retrospective cohort	Hospital setting	AKIN	age, history of AKI, vancomycin dose, concurrent receipt of nephrotoxins, concurrent receipt of vasopressors	88	Glycopeptide	AKI	Low
Craig, 2012 [15]	UK	Retrospective cohort	Hospital setting	Not stated	age, sex, operative procedure	200	Aminoglycoside	AKI	Low
Crellin, 2018 [16]	UK	Retrospective cohort	Multicentre Primary care	ICD-10 codes	sex, age, calendar period, chronic comorbidities, baseline renal function, history of renal or urological disease, and use of renin-angiotensin system blockers and potassium-sparing diuretics.	55,961	Cephalosporin	AKI	Low
Crellin, 2018 [16]	UK	Retrospective cohort	Multicentre Primary care	ICD-10 codes	sex, age, calendar period, chronic comorbidities, baseline renal function, history of renal or urological disease, and use of renin-angiotensin system blockers and potassium-sparing diuretics.	56,736	Nitrofurantoin	AKI	Low
Crellin, 2018 [16]	UK	Retrospective cohort	Multicentre Primary care	ICD-10 codes	sex, age, calendar period, chronic comorbidities, baseline renal function, history of renal or urological disease, and use of renin-angiotensin system blockers and potassium-sparing diuretics.	33,130	Quinolone	AKI	Low
Crellin, 2018 [16]	UK	Retrospective cohort	Multicentre Primary care	ICD-10 codes	sex, age, calendar period, chronic comorbidities, baseline renal function, history of renal or urological disease, and use of renin-angiotensin system blockers and potassium-sparing diuretics.	153,201	Trimethoprim	AKI	Low
Fraisse, 2014 [17]	France	Retrospective cohort	Hospital setting multicentre	NKF	age, sex, weight, concomitant use of other nephrotic drugs, types of infection	109	Aminoglycoside	AKI	Low
Fraisse, 2014 [17]	France	Retrospective cohort	Hospital setting multicentre	NKF	age, sex, weight, concomitant use of other nephrotic drugs, types of infection	45	Aminoglycoside	AKI	Low
Gandhi, 2013 [18]	Canada	Retrospective cohort	Population-based	ICD-10 codes	age, sex, chronic kidney disease, others	96,226	Macrolide	AKI	Low
Gandhi, 2013 [18]	Canada	Retrospective cohort	Population-based	ICD-10 codes	age, sex, chronic kidney disease, others	94,083	Macrolide	AKI	Low
Giri, 2016 [34]	India	Prospective cohort	Prospective cohort	Laboratory values	Overweight, age, sex, impaired renal status, pregnancy, immunocompromised	26	Aminoglycoside	AKI	High
Gyamlani, 2019 [19]	USA	Retrospective cohort	Hospital setting	CKD-EPI equation	age, sex, comorbidities, baseline eGFR, exposure to nephrotoxic medication	22,057	Glycopeptide	AKI	Low
Hall, 2014 [20]	USA	Retrospective cohort	Hospital setting	Laboratory values	age, sex, hospital stay, concomitant use of nephrotoxins	92	Glycopeptide	AKI	Low
Huang, 2018 [21]	China	Retrospective cohort	Hospital setting	AKIN	age, sex, type of infection, comorbidities, APACH II score	50	Glycopeptide	AKI	Low
Karino, 2014 [22]	Japan	Prospective cohort	Hospital setting	Categorical scale of mild, moderate, or serious (state recommended).	sex, age, underlying disease	22	Penicillin	AKI	Low
Li, 2015 [24]	Canada	Retrospective cohort	Population-based	ICD-10 codes	age, sex, baseline evidence of CKD, CVD, cancer, diabetes	52,518	Macrolide	AKI	Low
Li, 2015 [24]	Canada	Retrospective cohort	Population-based	ICD-10 codes	age, sex, baseline evidence of CKD, CVD, cancer, diabetes	51,523	Macrolide	AKI	Low
Liu, 2015 [23]	China	Retrospective cohort	Hospital setting	AKIN	age, sex, weight, comorbidities, concomitant use of ACEI, ARBs, NSAIDs, aminoglycosides, immunosuppressants	124	Glycopeptide	AKI	Low
Mizokami, 2013 [25]	Japan	Retrospective cohort	Hospital setting	Laboratory values	age, gender, infection severity, body weight, comorbidity index	94	Glycopeptide	AKI	Low
Morimoto, 2017 [32]	Japan	Prospective cohort	Hospital setting	KDIGO	Age, gender, concomitant antibiotics, BMI, BUN, severity of pneumonia	57	Penicillin	AKI	Moderate
Noh, 2019 [26]	South Korea	Retrospective cohort	Hospital setting	Laboratory values	age, sex, comorbidities (e.g., hypertension, etc.)	77	Antituberculosis drugs	DILI	Low
Ong, 2016 [27]	Singapore	Retrospective cohort	Hospital-based	KDIGO	age, sex, critical illness, comorbidities (e.g. diabetes, etc.), nephrotoxic medication	194	Aminoglycoside	AKI	Low
Ong, 2016 [27]	Singapore	Retrospective cohort	Hospital-based	KDIGO	age, sex, critical illness, comorbidities (e.g. diabetes, etc.), nephrotoxic medication	84	Aminoglycoside	AKI	Low
Pan, 2018 [39]	China	Retrospective cross-sectional	Hospital-based	KDIGO	age, gender, baseline serum creatinine, vasopressors, beta-blockers, furosemide, carbapenems, ICU admittance, etc.	647	Glycopeptide	AKI	Low
Pan, 2017 [28]	China	Retrospective cohort	Hospital-based	KDIGO	sex, age, baseline serum creatinine, the reason for vancomycin therapy, ICU admittance, etc.	279	Glycopeptide	AKI	Low
Paterson. 1998 [29]	Australia	Prospective cohort	Hospital-based	Laboratory values	age, duration of therapy, allopurinol use,	88	Aminoglycoside	AKI	Low
Raveh, 2002 [30]	Israel	Prospective cohort	Hospital-based	Laboratory values	age, gender, BMI, infectious diagnosis, diabetes mellitus, etc.	209	Aminoglycoside	AKI	Low
Sia, 2018 [35]	Australia	Retrospective cohort	Hospital based	KDIGO	Age, gender, treatment indicator, ICU admission	242	Aminoglycoside	AKI	Low

KEY: *AKI* Acute kidney injury, *DILI* Drug induced liver injury, *KDIGO* Kidney Disease: Improving Global Outcome, *AKIN* Acute Kidney Injury Network, *ICD-10* International Classification of Diseases version 10, *CKD-EPI* Chronic kidney Disease – Epidemiology collaborator, *NKF* National Kidney Foundation, *CVD* Cardiovarscular disease, *ACEI* Angiotensin converting enzyme Inhibitor, *ARB* Angiotensin II receptor blocker, *NSAID* Nonsteroidal anti-inflammatory drug, *ICU* Intensive care unit, *BMI* Body-Mass index

NOS risk of bias grades: low, moderate, high

### Characteristics of included studies

Out of the 29 studies included for qualitative analysis, eight were prospective cohort studies [12, 22, 29–31, 33, 34, 36] and the rest were all retrospective cohort studies. Most of the studies were hospital-based except four that were based on primary care data [11, 13, 16, 37] and three that were based on population data [18, 24, 36]. Six of the studies were carried out in the United Kingdom [11, 13, 15, 16, 37, 38], five from the United States of America [12, 14, 19, 20, 36], four from China [21, 23, 28, 39], three from Japan [22, 25, 32], two each from Australia [29, 35], Canada [18, 24] and India [31, 34], and one each from France [17], Iran [33], Israel [30], Singapore [27], and South Korea [26]. Out of the twenty-nine studies included for qualitative analysis, one reported on tendon rupture [36], three reported on drug-induced liver injury (DILI) [13, 26, 33], and the rest reported on acute kidney injury (AKI). After considering the classes of antimicrobials used, nine, eight and four studies reported on AKI due to aminoglycosides, glycopeptides, and macrolides, respectively. Cephalosporins, penicillin, quinolone, and trimethoprim had a couple of studies, each reporting on AKI, while nitrofurantoin had a single study reporting on AKI. Three studies were on antituberculosis antimicrobials, and they reported on DILI. One study on quinolones reported on tendon rupture. Table 1 below summarises the main characteristics of all the included studies.

### Risk of bias

The Newcastle-Ottawa Scale (NOS) [5] with slight modifications was used to assess the risk of bias for each of the included observational studies. This tool is well validated and commonly used for assessing the risk of bias for observational studies included in a systematic review [40]. In this systematic review, the modified tool is shown in additional file 4_doc1_appendices. Table 2 above summarises the risk of bias for each included observational study. All but two of the included cohort studies had a low risk of bias. The study by Morimoto et al. [32] had moderate risk of bias due to low score on selection criteria, while the study by Giri et al. [34] had high risk of bias due to low score on comparability.

**Table 2:**
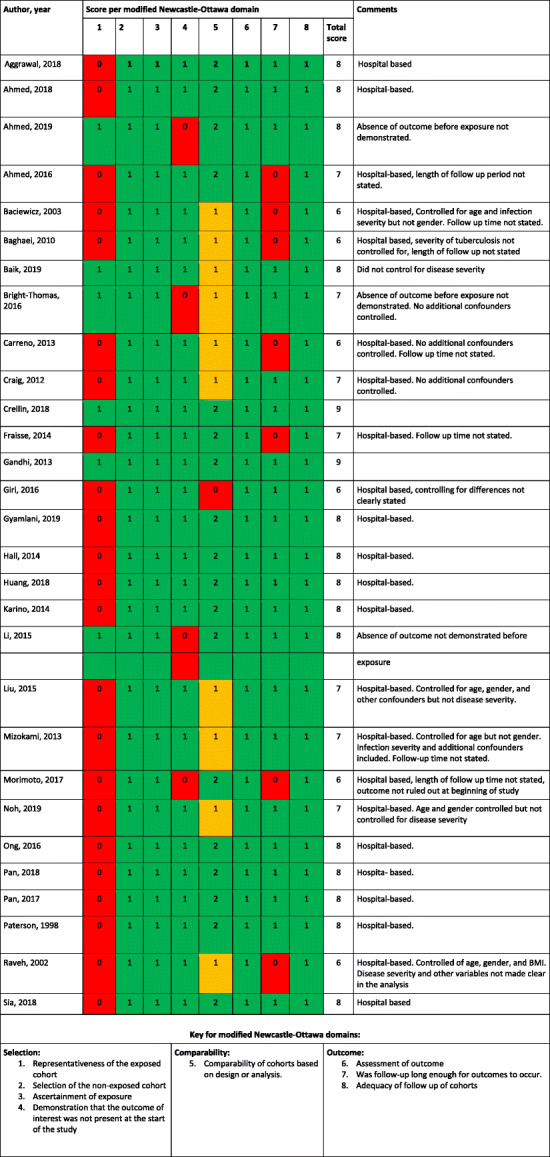
Quality assessment of included studies based on the modified Newcastle-Ottawa scoring system (summarised from additional file 3, document 1)

Key for modified Newcastle-Ottawa domains:

Selection:

1. Representativeness of the exposed cohort

2. Selection of the non-exposed cohort

3. Ascertainment of exposure

4. Demonstration that the outcome of interest was not present at the start of the study

Comparability:

5. Comparability of cohorts based on design or analysis.

Outcome:

6. Assessment of outcome

7. Was follow-up long enough for outcomes to occur.

8. Adequacy of follow up of cohorts

Red = Failed to satisify domain (Reduces quality)

Amber = Moderately satisfied domain (moderately affects quality)

Green = Satisfied domain (Increases quality)

### Outcomes

Out of the 29 studies included in this systematic review, two studies were not considered for meta-analysis due to their moderate or high risk of bias [32, 34]. A further two studies were not included in the meta-analysis of absolute risk because the researchers reported odds ratios only [16, 37]. However, the remaining twenty-five studies expressed the absolute risk of organ injury among exposed older adults. Eight studies [12, 15, 17, 27, 29, 30, 35, 38] reported absolute risk of AKI due to aminoglycoside exposure, with two antimicrobials reported in each of the studies by Fraisse et al. [17] and Ong et al. [27]. Eight studies reported absolute risk of AKI due to glycopeptide exposure [14, 19–21, 23, 25, 28, 39], and two studies reported the absolute risk of AKI due to macrolide exposure [18, 24] on two antimicrobials each. Two studies each on penicillin [11, 22], quinolones [16, 37], cephalosporins [16, 37], and trimethoprim [11, 16] also reported an absolute risk of AKI due to exposure to the respective antimicrobial. Only three studies [13, 26, 33] reported an absolute risk of DILI due to antituberculosis antimicrobials. Meta-analyses were done on absolute risks for AKI among older adults who received aminoglycoside, glycopeptide, or macrolide antimicrobials, and the respective attributable risk percentages were determined.

#### Amino glycopeptides

**Fig. 2:**
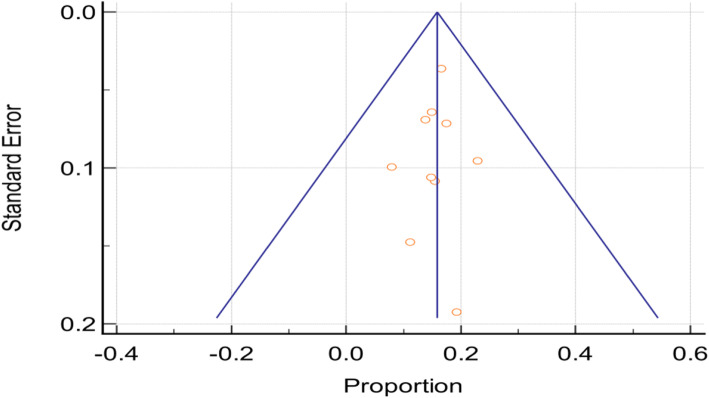
Funnel plot for studies included in the meta-analysis of the proportion of acute kidney injury among older adults prescribed aminoglycosides

**Fig. 3:**
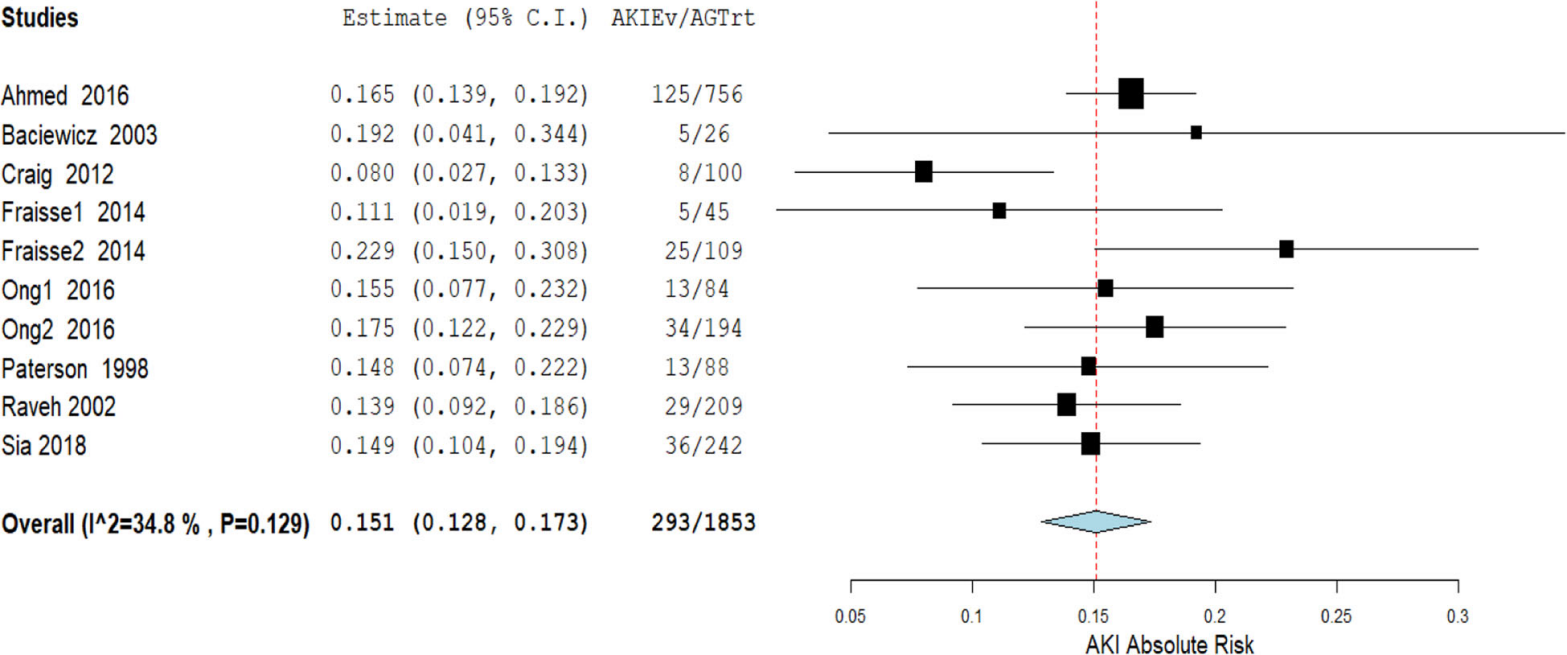
Meta-analysis of the proportion of acute kidney injury among older adults prescribed aminoglycosides. AKIEv = acute kidney injury cases. AGTrt = number exposed to aminoglycosides

A total of 1853 participants were exposed to aminoglycosides, and 293 of them were reported to have developed antimicrobial associated AKI across the eight studies. Figure 2 below, is the funnel plot of the included studies showing a very low degree of publication bias. The random-effects model was used to meta analyse the studies to establish the overall risk of AKI among older adults using aminoglycoside antimicrobials. Figure 3 below, is the forest plot of the meta-analysis and the studies show a high degree of homogeneity (I^2^ = 34.8%, *p* = 0.129). The overall absolute risk of AKI among older adults exposed to aminoglycosides was 15.1% (95% CI: 12.8–17.3%). This was significantly higher (*p* < 0.0001) than the average risk of AKI among adults of 18 years and above, following aminoglycoside antimicrobial exposure (10.5%; 95% CI: 10.1–10.8%) [41]. Therefore, the attributable risk per cent of AKI among older adults exposed to aminoglycosides was 30.5% (95% CI: 6.6–54.4%).

#### Glycopeptides

**Fig. 4:**
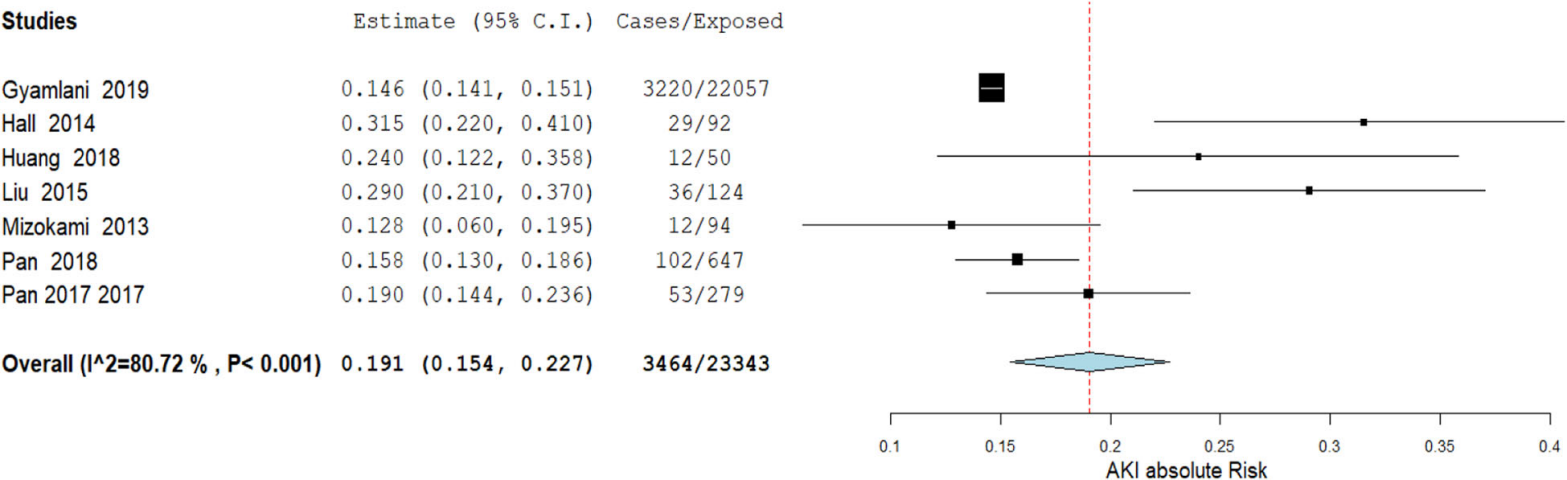
Meta-analysis of the proportion of acute kidney injury among older adults prescribed glycopeptides after removing the outlier

**Fig. 5:**
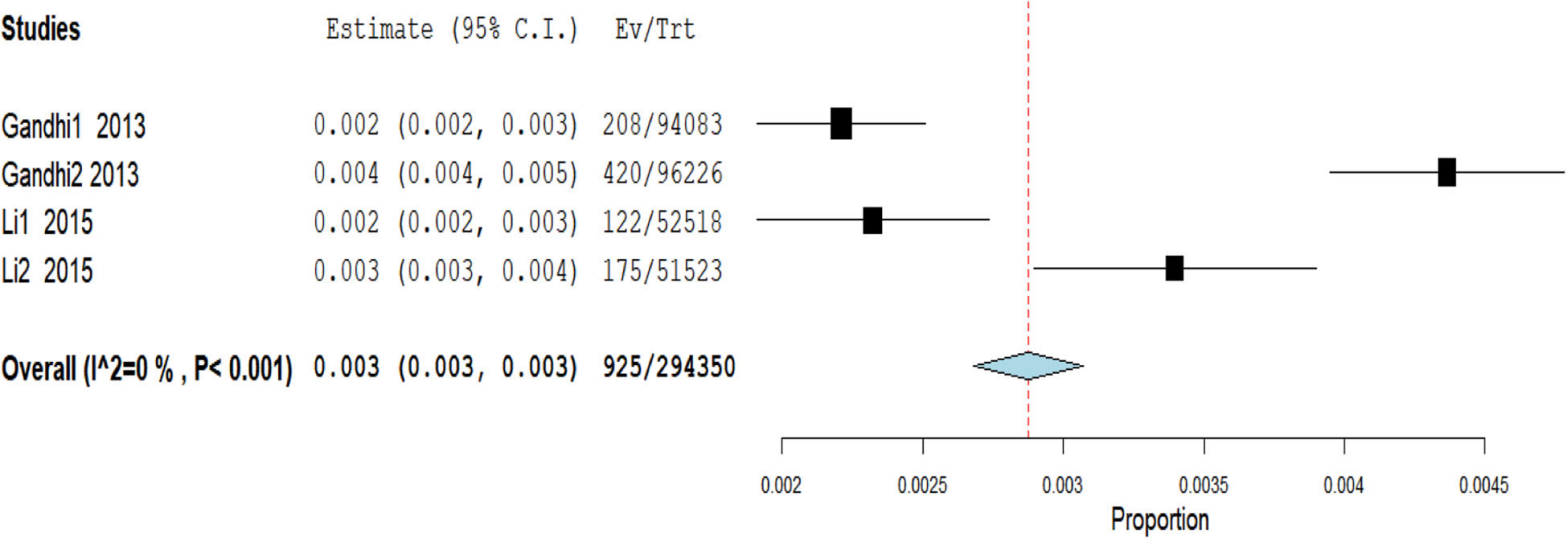
Meta-analysis of the proportion of acute kidney injury among older adults prescribed macrolides

A total of 23,431 participants were included in the eight studies that reported AKI due to glycopeptide exposure among older adults. Figure 4 (additional file 5, document 1) is a funnel plot showing the distribution of the studies when the standard error was plotted against the proportion of cases, and Fig. 5 (additional file 5, document 1) shows the forest plot before removing outliers, with an absolute risk of 21.4% (95% CI: 17.1–25.7%). After performing the leave-one-out meta-analysis, the overall absolute risk reduced significantly (*p* = 0.017) after excluding the study by Carreno et al. [14]. As shown in Fig. 6 (additional file 5, document 1), excluding other studies did not significantly impact the overall absolute risk of AKI. Figure 7 (additional file 5, document 1) shows the funnel plot following the outlier’s exclusion, and Fig. 4 shows the forest plot for a random-effects model for the studies after removing the outlier. In this systematic review, the overall absolute risk of AKI following the use of glycopeptides is, therefore, 19.1% (95% CI: 15.4–22.7%). There is no significant difference (*p* = 0.117) with the established risk of AKI among adults (18 years and above) on glycopeptide antimicrobial treatment (absolute risk = 18.7%; 95% CI: 15.6–21.7%) [42].

#### Macrolides

Only two studies reported on the risk of macrolide-associated AKI, but among them, a total of 294,350 participants were involved [18, 24]. Each of these two studies reported on two antimicrobials. The fixed-effects model was used to analyse these studies. Figure 5 is the forest plot summarising the combined outcome of these four studies. The overall risk of AKI among older adults exposed to macrolides was 0.3% (95% CI: 0.3–0.3%).

#### Drug-induced liver injury (DILI)

Only three studies, with 572 participants, reported antimicrobial-associated DILI [13, 26, 33]. In their study, Noh et al. [26] followed up 77 outpatients who were diagnosed and being treated for latent tuberculosis infection (LTBI), of which 14.3% (=11) had raised aminotransferases (the laboratory marker used to determine DILI) due to antituberculosis antimicrobials. All the participants were 65 years or above. Similarly, Bright-Thomas et al. [13] followed up 2070 adults (18 years and above) with active tuberculosis over 30 years (1981 to 2010). A subgroup of two hundred of these patients was 70 years old or more, hence included in this systematic review. Five per cent (*n* = 10) of these patients had raised aminotransferases, an indication of antimicrobial associated DILI. Baghaei et al. [33] carried out a prospective cohort study on risk of drug induced hepatitis among adult patients on anti-tuberculosis treatment. A subgroup of participants aged 65 years or above (*n* = 295) was considered for this systematic review, of which 16.6% (*n* = 49) had antimicrobial associated hepatitis. All three studies agreed that antimicrobial associated DILI increased with age.

#### Subgroup analysis

No studies included in this systematic review investigated the relative risk or odds ratio of organ injury associated with broad-spectrum antimicrobials compared to narrow-spectrum antimicrobials prescribed to older adults. However, three studies in one article [37] and another separate study [11] compared the odds of AKI among older adults when empirically prescribed different antimicrobials for urinary tract infection (UTI) with empirical prescription of nitrofurantoin. In another four studies published in one research paper [16], the odds of AKI among older adults prescribed different antimicrobials following UTI were compared with the odds following amoxicillin prescription. Another five studies each had data on long term compared to short term treatment and an age group comparison of those between 65 and 79 years versus those 80 years and above.

##### Organ injury following empirical antimicrobial prescription for UTI

Ahmed et al. [37] and Ahmed et al. [11] carried out several studies to determine the odds ratios of AKI when older adults have prescribed either cephalexin, co-amoxiclav, ciprofloxacin or trimethoprim when compared to nitrofurantoin for UTI. All exposure antimicrobials, except trimethoprim, had similar odds of causing UTI to nitrofurantoin among older adults. Trimethoprim was 1.9 times more likely to be associated with AKI when compared to nitrofurantoin (95% CI: 1.5–2.5). Crellin et al. [16] also reported the odds ratios of AKI among the older adults when cefalexin, ciprofloxacin, nitrofurantoin and trimethoprim were compared to amoxicillin. The odds of AKI associated with nitrofurantoin or cefalexin was not significantly different to that of amoxicillin among the older adults (OR = 0.89; 95% CI: 0.65–1.24, and OR = 1.01, 95% CI: 0.74–1.37, respectively). Ciprofloxacin was 1.48 (95% CI: 1.03–2.13) times, and trimethoprim was 1.72 (95% CI: 1.31–2.24) times more likely to be associated with AKI among older adults when compared to amoxicillin when prescribed for UTI.

##### Organ injury following treatment duration

Among the five studies that included duration of treatment as part of their analysis, three of them [27, 29, 30] compared up to 7 days of treatment with aminoglycosides versus more than 7 days, while the other two studies [19, 20] compared short and long term treatments with glycopeptides. In the study by Paterson et al. [29], there was a strong association of AKI among the patients who received an aminoglycoside for more than 7 days, with a risk ratio of 7.5 (95% CI: 2.7–20.5, *p* = 0.0001), when compared to those treated for seven or fewer days. Contrary, in both of their studies, Ong et al. [27] and Raveh et al. [30] found no association of risk of AKI among those who received an aminoglycoside for more than 7 days, when compared to 7 or fewer days of treatment, with relative risks of 0.99 (95% CI: 0.58–1.68) and 1.35 (95% CI: 0.68–2.68) respectively. Similarly, Gyamlani et al. [19] and Hall et al. [20] did not find any association between the risk of AKI among those who received long term glycopeptide treatment compared to short term treatment.

##### Organ injury according to age group

Three of the five studies determined the association of AKI due to glycopeptide treatment among those at least 80 years when compared to those between 65 and 79 years [14, 20, 28]. According to Pan et al. [28], there was a significant association of AKI among those at least 80 years old and on glycopeptide therapy when compared to those between 65 and 79 years of age, with a relative risk of 2.3 (95% CI: 1.5–3.8, *p* = 0.0004). However, the studies by Hall et al. [20] and Carreno et al. [14] did not show any significant association of older age with AKI due to glycopeptide therapy. Similarly, both Bright-Thomas et al. [13] and Noh et al. [26] did not find any significant association of older age with DILI due to antituberculosis antimicrobials, with respective relative risks of 1.52 (95% CI: 0.44–5.19) and 1.18 (95% CI: 0.18–7.75).

### Heterogeneity and publication bias

The included aminoglycoside studies were tested for heterogeneity and the Q-test was 11.59 (*p* = 0.2376), and I^2^ was 22.32% (95% CI: 0.00–62.07%). Figure 2 (above) is the funnel plot of the included studies showing a central and uniform distribution of both small and large studies around the mean, indicating a low degree of publication bias. The Egger’s regression test intercept was − 0.3564 (95% CI: − 2.341 to 1.628).

The funnel plot for studies on glycopeptide exposure (Fig. 4, additional file 5, document 1) was skewed to the right, with one study possibly an outlier. Figure 5 (additional file 5, document 1) is the forest plot before sensitivity analysis, with an absolute risk of 21.4% (95% CI: 17.1–25.7%). After performing the leave-one-out meta-analysis, the overall absolute risk reduced significantly (*p* = 0.017) after excluding the study by Carreno et al. (16). As shown in Fig. 6 (additional file 5, document 1), excluding other studies did not significantly impact the overall absolute risk of AKI. The Q – test was 39.08 (*p* < 0.0001) and the I^2^ test was 84.65% (95% CI: 70.15–92.10%). The funnel plot (Fig. 7, additional file 5, document 1) was skewed, and Egger’s regression test intercept was 2.2664 (*p* = 0.0340).

## Discussion

This is the first systematic review with meta-analysis to investigate kidney, liver, and tissue injury associated with antimicrobial exposure among older adults 65 years and above. This systematic review’s primary outcome of interest was to determine the overall risk of organ injury among older adults who received antimicrobial therapy. Most of the studies included in our systematic review expressed the risk of kidney injury following exposure to aminoglycoside or glycopeptide antimicrobials.

### Antimicrobial exposure and risk of organ injury

In our systematic review, older adults had a 15.1% (95% CI: 12.8–17.3%) risk of developing AKI following exposure to aminoglycosides. Several reviews and original research studies have found a wide variation of AKI risk among patients prescribed these antimicrobials. Selby et al. [43] found a 24.4% risk of AKI among hospitalised patients associated with increased mortality. Oliveira et al. [44] found the prevalence of AKI among intensive care unit patients prescribed aminoglycosides to be as high as 58% and associated with increased mortality. On the lower end, Paquette et al. [45] found the risk of AKI from aminoglycoside exposure to be 12% after excluding children, seriously ill patients, and patients treated for less than 5 days. In a large study of 8270 participants, Fuhrman et al. [46] found the incidence of AKI associated with aminoglycosides to be only 4 %. Oliveira et al. [44] argued that this huge variation in prevalence is due to varying study population characteristics. However, the majority of published literature estimates the prevalence to be 10 to 20%. In their systematic review and meta-analysis, Hayward et al. [41] found a 10.5% incidence of AKI among adults of 18 years to 95 years of age, from a total of 24,107 non-intensive care patients. Using this systematic review as our reference, we concluded that older adults are at significantly higher risk of AKI when prescribed aminoglycosides (*p* < 0.0001), with attributable risk from 6.6 to 54.4%.

In our systematic review, glycopeptides were associated with a 19.1% (95% CI: 15.4–22.7%) risk of AKI among older adults. Gyamlani et al. [19] noted that AKI incidence due to glycopeptide exposure varies between 5 and 43%, depending on population and baseline risk factors. In their study, Gyamlani et al. found a 10.4% incidence of AKI among patients prescribed glycopeptides. In another study, Fuhrman et al. [46] found out that 17% of adults prescribed glycopeptides developed AKI during or post-exposure. In their systematic review and meta-analysis, Sinha Ray et al. [42] found the incidence of AKI among adults 18 years and above to be 18.7% (95% CI: 15.6–21.7%). Using this study as our baseline, there was no significant difference with older adults’ findings (*p* = 0.117). We, therefore, concluded that older adults of 65 years and above have a similar risk of developing AKI when prescribed glycopeptides as the general adult population of 18 years and above.

Only 0.3% of AKI was associated with macrolides to older adults in our systematic review. This is a rare outcome; therefore, there are not many studies done to explore this relationship. Similarly, we only found three studies that reported antimicrobial associated DILI among older adults. The number of studies was too small to perform a meaningful meta-analysis. However, the three studies had an average of 12.3% risk of antimicrobial associated DILI among older adults. Although fewer studies report antimicrobial-associated DILI among older adults, several studies have been reported on the general adult population. In a Swedish study, 27% of DILI was attributed to antimicrobials [47]. Similar studies were also done in Spain [48], India [49] and the United States of America [50] and found 32, 65, and 45% of DILI associated with antimicrobials, respectively. Several antimicrobials have been implicated, but most commonly were amoxicillin-clavulanic acid [48, 50], erythromycin [51] and nitrofurantoin [50].

### Antimicrobial exposure and risk of organ injury by the duration

In our systematic review, the three studies that reported on the association of AKI with aminoglycoside treatment duration did not agree on their findings. Paterson et al. [29] found a relative risk ratio of 7.5 when patients were prescribed aminoglycosides for more than 7 days compared to those less than 7 days. However, Raveh et al. [30] reported an increased risk of AKI when aminoglycoside treatment was prolonged to 11 days and above. However, Selby et al. [43] did not find any association between AKI and treatment duration.

### Antimicrobial exposure and risk of organ injury by age

We did not find any studies that assessed the association of aminoglycoside prescription with AKI by age group. However, some authors have found an increased risk of AKI with age from the general adult population [43]. Only one out of the three studies in our systematic review showed a significant association of age with AKI following glycopeptide prescription to older adults [28]. In their systematic review, van Hal et al. [52] found out that AKI risk following glycopeptide prescription increases with age if patients are exposed for more than 7 days.

### Heterogeneity and publication bias

In our systematic review, we used the random-effects model to summarise the risk of AKI among older adults prescribed aminoglycosides and glycopeptides. Several authors, for example, Ioannidis [53] and Huedo-Medina et al. [54], decide on whether to use a fixed-effect or random-effects model based on heterogeneity results. However, we chose our model before heterogeneity testing, based on recommendations by Borenstein et al. [55] and further supported by Spinelli and Pandis [56]. Our studies were drawn from across the globe and were carried out under different conditions, so we assumed they were not identical. In this instance, heterogeneity testing helped us determine whether these studies have enough in common to be meta-analysed [56]. We also used the funnel plot [7] and Egger’s regression test [8] to assess our studies’ publication bias. We meta-analysed nine studies on aminoglycosides and seven studies on glycopeptides.

### Strengths of our study

The study focused on older adults aged 65 and above, who are usually underrepresented in randomised controlled trials. Real-world data, which is more representative of the older adults prescribed antimicrobials, was used. The quality of each included study was thoroughly assessed on representativeness on study participants, ascertainment of exposure of interest, absence of outcome of interest at the beginning of the study, control of possible confounders including age, gender, and severity of the disease, outcome assessment, and adequacy of follow up for outcome to occur.

Most of the included studies shared some common strengths. All the studies considered a wide range of possible confounders and adjusted for them accordingly in their analyses. Well known and validated tools were used to ascertain the presence or absence of outcomes. For example, the international classification of diseases version 10 (ICD-10) was used by authors whose study settings were primary care or population-based [11, 16, 18, 24, 37]. Most hospital-based studies used tools that relied on laboratory markers for organ injury, such as a two-fold rise in serum creatinine or doubling of liver transaminases. Some of the tools used include the acute kidney injury network (AKIN) [14, 21, 38] and kidney disease, improving global outcome (KDIGO) [27, 28, 39]. Only three studies [13, 24, 37] did not describe how they determined the absence of outcome of interest at the beginning of their studies.

### Limitations of our study

Despite their strengths, the included studies in this systematic review also came with their weaknesses. All the included studies, except five [13, 16, 18, 24, 37], were hospital-based. The general major drawback of using hospitalised patients is the generalisability of the findings [57]. Most of our data were obtained from subgroups embedded in larger studies. This led to our data lacking detailed information, for example, gender differences among the older adults, since we relied heavily on limited results from tables or otherwise.

## Conclusion

In conclusion, the findings from this systematic review and meta-analysis suggest that there is a significantly increased risk of AKI among older adults when compared to the general adult population following exposure to aminoglycosides. Our results indicated that the risk of AKI in older adults is 15.1% (95% CI: 12.8–17.3). The risk of AKI following exposure to glycopeptides for older adults is similar to that previously found in the general adult population. We did not get enough studies to determine whether age or duration of treatment influences the risk of AKI among older adults.

## Supplementary Information

**Additional file 1.** Risk of antimicrobial-associated organ injury among older adults: A systematic Review and Meta-Analysis.

**Additional file 2.** Risk factors for nephrotoxicity in elderly patients receiving once-daily aminoglycosides.

**Additional file 3.** Study appraisals – Risk of Bias assessment using Newcastle-Ottawa scale.

**Additional file 4: Appendix 1.** Modified Newcastle-Ottawa Scale: Cohort Studies tool.

**Additional file 5.** Funnel plots and leave-one-meta-analysis figures.

## Data Availability

All data generated or analysed during this study are included in this published article (and its supplementary information files).

## Abbreviations

ACEI: Angiotensin converting enzyme Inhibitor
AKI: Acute kidney injury
AKIN: Acute Kidney Injury Network
ARB: Angiotensin II receptor blocker
CKD-EPI: Chronic kidney Disease – Epidemiology collaborator
DILI: Drug-induced liver injury
DILI: Drug induced liver injury
ICD-10: International Classification of Diseases version 10
ICU: Intensive care unit; BMI- Body-Mass index
KDIGO: Kidney Disease: Improving Global Outcome
LTBI: Latent tuberculosis infection
NKF: National Kidney Foundation
CVD: Cardiovarscular disease
NSAID: Nonsteroidal anti-inflammatory drug
UTI: Urinary tract infection
