# Supplementary material for: Risk of antimicrobial-associated organ injury among the older adults: a systematic review and meta-analysis
Source: BMC Geriatr. 2021 Nov 1;21:617. doi: 10.1186/s12877-021-02512-3 (PMC8561875; doi:10.1186/s12877-021-02512-3)
Supplement: Supplementary file 1 — Additional file 1. Risk of antimicrobial-associated organ injury among older adults: A systematic Review and Meta-Analysis. [file 12877_2021_2512_MOESM1_ESM.docx]

**Risk of antimicrobial-associated organ injury among older adults: A systematic Review and Meta-Analysis.**

**Database search strategy**

Searches were conducted in PubMed, Embase.com, Web of Science core collection, Web of Science BIOSIS citation index, Scopus, Cochrane Central Register of Controlled Trials, ProQuest, and PsycINFO databases, using key words in titles and abstracts, and using MeSH terms. For each database, the search comprised of three themes including the exposure, outcome and population of interest. The antimicrobials, organ injury (kidney, liver, and tissue), and older adults were used respectively, with their synonyms. The table below shows how the searches were conducted in each of the databases.

| **Database** | **Search #** | **Search query** | **Results** |
| --- | --- | --- | --- |
| **PubMed** | #1 | "anti-bacterial agent*"[Title/Abstract] OR "aminoglycoside*"[Title/Abstract] OR "beta lactam*"[Title/Abstract] OR "penicillin*"[Title/Abstract] OR "monobactam*"[Title/Abstract] OR "carbapenem*"[Title/Abstract] OR "cephalosporin*"[Title/Abstract] OR "fluoroquinolone*"[Title/Abstract] OR "glycopeptide*"[Title/Abstract] OR "lipoglycopeptide*"[Title/Abstract] OR "macrolide*"[Title/Abstract] OR "oxazolidinone*"[Title/Abstract] OR "rifamycin*"[Title/Abstract] OR "sulfonamide*"[Title/Abstract] OR "streptogramin*"[Title/Abstract] OR "tetracycline*"[Title/Abstract] OR "lincosamide*"[Title/Abstract] OR "Chloramphenicol"[Title/Abstract] OR "Daptomycin"[Title/Abstract] OR "Fosfomycin"[Title/Abstract] OR "Metronidazole"[Title/Abstract] OR "Mupirocin"[Title/Abstract] OR "Nitrofurantoin"[Title/Abstract] OR "Anti-Bacterial Agents"[MeSH Terms] OR "Aminoglycosides"[MeSH Terms] OR "beta-Lactams"[MeSH Terms] OR "Fluoroquinolones"[MeSH Terms] OR "Glycopeptides"[MeSH Terms] OR "Macrolides"[MeSH Terms] OR "Oxazolidinones"[MeSH Terms] OR "Rifamycins"[MeSH Terms] OR "Sulfanilamides"[MeSH Terms] OR "Streptogramins"[MeSH Terms] OR "Tetracyclines"[MeSH Terms] OR "Lincosamides"[MeSH Terms] OR "Chloramphenicol"[MeSH Terms] OR "Daptomycin"[MeSH Terms] OR "Fosfomycin"[MeSH Terms] OR "Metronidazole"[MeSH Terms] OR "Mupirocin"[MeSH Terms] OR "Nitrofurantoin"[MeSH Terms] | 917,154 |
|  | #2 | "acute kidney injur*"[Title/Abstract] OR "acute renal injur*"[Title/Abstract] OR "acute renal insufficienc*"[Title/Abstract] OR "acute kidney insufficienc*"[Title/Abstract] OR "acute kidney failure*"[Title/Abstract] OR "acute renal failure*"[Title/Abstract] OR "chemically induced liver toxicit*"[Title/Abstract] OR "chemically induced liver toxicit*"[Title/Abstract] OR "drug induced acute liver injur*"[Title/Abstract] OR "drug induced acute liver injur*"[Title/Abstract] OR "toxic hepatitis"[Title/Abstract] OR "toxic hepatitides"[Title/Abstract] OR "drug induced liver disease*"[Title/Abstract] OR "drug induced liver disease*"[Title/Abstract] OR "drug induced liver injur*"[Title/Abstract] OR "drug induced liver injur*"[Title/Abstract] OR "drug-induced hepatitides"[Title/Abstract] OR "drug-induced hepatitides"[Title/Abstract] OR "drug-induced hepatitis"[Title/Abstract] OR "drug-induced hepatitis"[Title/Abstract] OR "tendon injur*"[Title/Abstract] OR "acute kidney injury/drug effects"[MeSH Terms] OR "chemical and drug induced liver injury/drug effects"[MeSH Terms] OR "tendon injuries/adverse effects"[MeSH Terms] OR "tendon injuries/toxicity"[MeSH Terms] | 63,229 |
|  | #3 | "older adult*"[Title/Abstract] OR "over 65"[Title/Abstract] OR "elderly"[Title/Abstract] OR "over 80"[Title/Abstract] OR "Aged"[Title/Abstract] OR "elderly"[Title/Abstract] OR "Aged"[MeSH Terms] OR "aged, 80 and over"[MeSH Terms] | 3,747,415 |
|  | **#4** | **#1 AND #2 AND #3** | **1,164** |
| **Embase.com** | #1 | 'anti-bacterial agent*':ti,ab,kw OR aminoglycoside*:ti,ab,kw OR 'beta-lactam*':ti,ab,kw OR penicillin*:ti,ab,kw OR monobactam*:ti,ab,kw OR carbapenem*:ti,ab,kw OR cephalosporin*:ti,ab,kw OR fluoroquinolone*:ti,ab,kw OR glycopeptide*:ti,ab,kw OR lipoglycopeptide*:ti,ab,kw OR macrolide*:ti,ab,kw OR oxazolidinone*:ti,ab,kw OR rifamycin*:ti,ab,kw OR sulfonamide*:ti,ab,kw OR streptogramin*:ti,ab,kw OR tetracycline*:ti,ab,kw OR lincosamide*:ti,ab,kw OR chloramphenicol:ti,ab,kw OR daptomycin:ti,ab,kw OR fosfomycin:ti,ab,kw OR metronidazole:ti,ab,kw OR mupirocin:ti,ab,kw OR nitrofurantoin:ti,ab,kw OR 'antibiotic agent'/exp OR 'aminoglycoside antibiotic agent'/exp OR 'beta lactam antibiotic'/exp OR 'quinoline derived antiinfective agent'/exp OR 'polypeptide antibiotic agent'/exp OR 'macrolide'/exp OR 'oxazolidinone'/exp OR 'rifamycin'/exp OR 'sulfanilamide derivative'/exp OR 'streptogramin derivative'/exp OR 'tetracycline derivative'/exp OR 'lincosamide'/exp OR 'chloramphenicol derivative'/exp OR 'daptomycin'/exp OR 'fosfomycin'/exp OR 'metronidazole'/exp OR 'pseudomonic acid'/exp OR 'nitrofurantoin'/exp | 1,826,825 |
|  | #2 | 'acute kidney injur*':ti,ab,kw OR 'acute renal injur*':ti,ab,kw OR 'acute renal insufficienc*':ti,ab,kw OR 'acute kidney insufficienc*':ti,ab,kw OR 'acute kidney failure*':ti,ab,kw OR 'acute renal failure*':ti,ab,kw OR 'chemically-induced liver toxicit*':ti,ab,kw OR 'chemically induced liver toxicit*':ti,ab,kw OR 'drug-induced acute liver injur*':ti,ab,kw OR 'drug induced acute liver injur*':ti,ab,kw OR 'toxic hepatitis':ti,ab,kw OR 'toxic hepatitides':ti,ab,kw OR 'drug-induced liver disease*':ti,ab,kw OR 'drug induced liver disease*':ti,ab,kw OR 'drug-induced liver injur*':ti,ab,kw OR 'drug induced liver injur*':ti,ab,kw OR 'drug induced hepatitides':ti,ab,kw OR 'drug-induced hepatitides':ti,ab,kw OR 'drug-induced hepatitis':ti,ab,kw OR 'drug induced hepatitis':ti,ab,kw OR 'tendon injur*':ti,ab,kw OR 'acute kidney failure'/exp OR 'drug-induced liver injury'/exp OR 'acute drug induced hepatitis'/exp OR 'chemical and drug induced liver injury'/exp OR 'tendon injury'/exp OR 'tendon rupture'/exp OR 'flexor tendon injury'/exp OR 'achilles tendon rupture'/exp | 153,449 |
|  | #3 | 'older adult*':ti,ab,kw OR 'over 65':ti,ab,kw OR 'elderly':ti,ab,kw OR 'over 80':ti,ab,kw OR aged:ti,ab,kw OR elderly:ti,ab,kw OR 'aged'/exp OR 'very elderly'/exp | 4.004,436 |
|  | **#4** | **#1 AND #2 AND #3** | **4,880** |
| **Web of Science Core collection** | #1 | TS=("Anti-Bacterial agent*" OR Aminoglycoside* OR "beta-lactam*" OR Penicillin* OR Monobactam* OR Carbapenem* OR Cephalosporin* OR Fluoroquinolone* OR Glycopeptide* OR lipoglycopeptide* OR Macrolide* OR Oxazolidinone* OR Rifamycin* OR Sulfonamide* OR Streptogramin* OR Tetracycline* OR lincosamide* OR chloramphenicol OR daptomycin OR Fosfomycin OR metronidazole OR mupirocin OR nitrofurantoin)  *Indexes=SCI-EXPANDED, SSCI, A&HCI, CPCI-S, CPCI-SSH, BKCI-S, BKCI-SSH, ESCI, CCR-EXPANDED, IC Timespan=All years* | 280,785 |
|  | #2 | TS=(“acute kidney injur*” OR “acute renal injur*” OR “acute renal insufficienc*” OR “acute kidney insufficienc*” OR “acute kidney failure*” OR “acute renal failure*” OR “chemically-induced liver toxicit*” OR “chemically induced liver toxicit*” OR “drug-induced acuted liver injur*” OR “drug induced acute liver injur*” OR “toxic hepatitis” OR “toxic hepatitides” OR “drug-induced liver disease*” OR “drug induced liver disease*” OR “drug-induced liver injur*” OR “drug induced liver injur*” OR “drug induced hepatitides” OR “drug-induced hepatitides” OR “drug-induced hepatitis” OR “drug induced hepatitis” OR “tendon injur*” )  *Indexes=SCI-EXPANDED, SSCI, A&HCI, CPCI-S, CPCI-SSH, BKCI-S, BKCI-SSH, ESCI, CCR-EXPANDED, IC Timespan=All years.* | 71,620 |
|  | #3 | TS=(“older adult*” OR “over 65” OR “elderly” OR “over 80” OR aged OR elderly)  *Indexes=SCI-EXPANDED, SSCI, A&HCI, CPCI-S, CPCI-SSH, BKCI-S, BKCI-SSH, ESCI, CCR-EXPANDED, IC Timespan=All years.* | 3,771,196 |
|  | **#4** | **#1 AND #2 AND #3** | **250** |
| **Web of Science BIOSIS citation index** | #1 | TS=(“Anti-Bacterial agent*” OR Aminoglycoside* OR “beta-lactam*” OR Penicillin* OR Monobactam* OR Carbapenem* OR Cephalosporin* OR Fluoroquinolone* OR Glycopeptide* OR lipoglycopeptide* OR Macrolide* OR Oxazolidinone* OR Rifamycin* OR Sulfonamide* OR Streptogramin* OR Tetracycline* OR lincosamide* OR chloramphenicol OR daptomycin OR Fosfomycin OR metronidazole OR mupirocin OR nitrofurantoin)  *Indexes=BCI Timespan=All years* | 275,428 |
|  | #2 | TS=(“acute kidney injur*” OR “acute renal injur*” OR “acute renal insufficienc*” OR “acute kidney insufficienc*” OR “acute kidney failure*” OR “acute renal failure*” OR “chemically-induced liver toxicit*” OR “chemically induced liver toxicit*” OR “drug-induced acuted liver injur*” OR “drug induced acute liver injur*” OR “toxic hepatitis” OR “toxic hepatitides” OR “drug-induced liver disease*” OR “drug induced liver disease*” OR “drug-induced liver injur*” OR “drug induced liver injur*” OR “drug induced hepatitides” OR “drug-induced hepatitides” OR “drug-induced hepatitis” OR “drug induced hepatitis” OR “tendon injur*” )  *Indexes=BCI Timespan=All years* | 42,473 |
|  | #3 | TS=(“older adult*” OR “over 65” OR “elderly” OR “over 80” OR aged OR elderly)  *Indexes=BCI Timespan=All years* | 3,267,436 |
|  | **#4** | **#1 AND #2 AND #3** | **301** |
| **Scopus** | #1 | TITLE-ABS-KEY (“anti-bacterial AND agent*” OR aminoglycoside* OR “beta-lactam*”  OR  penicillin*  OR  monobactam*  OR  carbapenem*  OR  cephalosporin*  OR  fluoroquinolone*  OR  glycopeptide*  OR  lipoglycopeptide*  OR  macrolide*  OR  oxazolidinone*  OR  rifamycin*  OR  sulfonamide*  OR  streptogramin*  OR  tetracycline*  OR  lincosamide*  OR  chloramphenicol  OR  daptomycin  OR  fosfomycin  OR  metronidazole  OR  mupirocin  OR  nitrofurantoin ) | 774,212 |
|  | #2 | TITLE-ABS-KEY ( "acute kidney injur*"  OR  "acute renal injur*"  OR  "acute renal insufficienc*"  OR  "acute kidney insufficienc*"  OR  "acute kidney failure"  OR  "acute renal failure"  OR  "chemically-induced liver toxicity"  OR  "chemically induced liver toxicity"  OR  "drug-induced acute liver injury"  OR  "drug induced acute liver injury"  OR  "toxic hepatitis"  OR  "toxic hepatitides"  OR  "drug-induced liver disease"  OR  "drug induced liver disease"  OR  "drug-induced liver injury"  OR  "drug induced liver injury"  OR  "drug induced hepatitides"  OR  "drug-induced hepatitides"  OR  "drug-induced hepatitis"  OR  "drug induced hepatitis"  OR  "tendon injury" ) | 136,781 |
|  | #3 | TITLE-ABS-KEY ( "older adult*"  OR  "over 65"  OR  "elderly"  OR  "over 80"  OR  aged  OR  elderly ) | 5,655,933 |
|  | **#4** | **#1 AND #2 AND #3** | **2901** |
| **Cochrane Central Register of Controlled Trials** | #1 | (‘Anti-Bacterial agent*’ OR Aminoglycoside* OR ‘beta-lactam*’ OR Penicillin* OR Monobactam* OR Carbapenem* OR Cephalosporin* OR Fluoroquinolone* OR Glycopeptide* OR lipoglycopeptide* OR Macrolide* OR Oxazolidinone* OR Rifamycin* OR Sulfonamide* OR Streptogramin* OR Tetracycline* OR lincosamide* OR chloramphenicol OR daptomycin OR Fosfomycin OR metronidazole OR mupirocin OR nitrofurantoin):ti,ab,kw | 29298 |
|  | #2 | MeSH descriptor: [anti-bacterial agents] explode all trees | 12389 |
|  | #3 | MeSH descriptor: [aminoglycosides] explode all trees | 8929 |
|  | #4 | MeSH descriptor: [beta-lactam] explode all trees | 9632 |
|  | #5 | MeSH descriptor: [fluoroquinolones] explode all trees | 3721 |
|  | #6 | MeSH descriptor: [glycopeptides] explode all trees | 2089 |
|  | #7 | MeSH descriptor: [macrolides] explode all trees | 9311 |
|  | #8 | MeSH descriptor: [oxazolidinone] explode all trees | 787 |
|  | #9 | MeSH descriptor: [rifamycin] explode all trees | 1568 |
|  | #10 | MeSH descriptor: [sulfonamides] explode all trees | 13656 |
|  | #11 | MeSH descriptor: [streptogramins] explode all trees | 25 |
|  | #12 | MeSH descriptor: [tetracyclines] explode all trees | 2574 |
|  | #13 | MeSH descriptor: [lincosamides] explode all trees | 928 |
|  | #14 | MeSH descriptor: [chloramphenicol] explode all trees | 312 |
|  | #15 | MeSH descriptor: [daptomycin] explode all trees | 97 |
|  | #16 | MeSH descriptor: [fosfomycin] explode all trees | 136 |
|  | #17 | MeSH descriptor: [metronidazole] explode all trees | 2338 |
|  | #18 | MeSH descriptor: [mupirocin] explode all trees | 224 |
|  | #19 | MeSH descriptor: [nitrofurantoin] explode all trees | 162 |
|  | **#20** | #1 OR #2 OR #3 OR #4 OR #5 OR #6 OR #7 OR #8 OR #9 OR #10 OR #11 OR #12 OR #13 OR #14 OR #15 OR #16 OR #17 OR #18 OR #19 | 58815 |
|  | #21 | (‘acute kidney injur*’ OR ‘acute renal injur*’ OR ‘acute renal insufficienc*’ OR ‘acute kidney insufficienc*’ OR ‘acute kidney failure*’ OR ‘acute renal failure*’ OR ‘chemically-induced liver toxicit*’ OR ‘chemically induced liver toxicit*’ OR ‘drug-induced acute liver injur*’ OR ‘drug induced acute liver injur*’ OR ‘toxic hepatitis’ OR ‘toxic hepatitides’ OR ‘drug-induced liver disease*’ OR ‘drug induced liver disease*’ OR ‘drug-induced liver injur*’ OR ‘drug induced liver injur*’ OR ‘drug induced hepatitides’ OR ‘drug-induced hepatitides’ OR ‘drug-induced hepatitis’ OR ‘drug induced hepatitis’ OR ‘tendon injur*’):ti,ab,kw | 17877 |
|  | #22 | MeSH descriptor: [acute kidney injury] explode all trees | 1516 |
|  | #23 | MeSH descriptor: [chemical and drug induced liver inury] explode all trees | 323 |
|  | #24 | MeSH descriptor: [tendon injuries] explode all trees | 1623 |
|  | **#25** | #21 OR #22 OR #23 OR #24 | 19028 |
|  | #26 | (‘older adult*’ OR ‘over 65’ OR ‘elderly’ OR ‘over 80’ OR aged OR elderly):ti,ab,kw | 757028 |
|  | #27 | MeSH descriptor: [aged] explode all trees | 211493 |
|  | #28 | MeSH descriptor: [aged, 80 and over] explode all trees | 53973 |
|  | **#29** | #26 OR #27 OR #28 | 757028 |
|  | **#30** | **#20 AND #25 AND #29** | **817** |
| **ProQuest** | #1 | ab(“Anti-Bacterial agent*” OR Aminoglycoside* OR “beta-lactam*” OR Penicillin* OR Monobactam* OR Carbapenem* OR Cephalosporin* OR Fluoroquinolone* OR Glycopeptide* OR lipoglycopeptide* OR Macrolide* OR Oxazolidinone* OR Rifamycin* OR Sulfonamide* OR Streptogramin* OR Tetracycline* OR lincosamide* OR chloramphenicol OR daptomycin OR Fosfomycin OR metronidazole OR mupirocin OR nitrofurantoin) OR ti(“Anti-Bacterial agent*” OR Aminoglycoside* OR “beta-lactam*” OR Penicillin* OR Monobactam* OR Carbapenem* OR Cephalosporin* OR Fluoroquinolone* OR Glycopeptide* OR lipoglycopeptide* OR Macrolide* OR Oxazolidinone* OR Rifamycin* OR Sulfonamide* OR Streptogramin* OR Tetracycline* OR lincosamide* OR chloramphenicol OR daptomycin OR Fosfomycin OR metronidazole OR mupirocin OR nitrofurantoin) | 24695 |
|  | #2 | ab(“acute kidney injur*” OR “acute renal injur*” OR “acute renal insufficienc*” OR “acute kidney insufficienc*” OR “acute kidney failure*” OR “acute renal failure*” OR “chemically-induced liver toxicit*” OR “chemically induced liver toxicit*” OR “drug-induced acute liver injur*” OR “drug induced acute liver injur*” OR “toxic hepatitis” OR “toxic hepatitides” OR “drug-induced liver disease*” OR “drug induced liver disease*” OR “drug-induced liver injur*” OR “drug induced liver injur*” OR “drug induced hepatitides” OR “drug-induced hepatitides” OR “drug-induced hepatitis” OR “drug induced hepatitis” OR “tendon injur*” ) OR ti(“acute kidney injur*” OR “acute renal injur*” OR “acute renal insufficienc*” OR “acute kidney insufficienc*” OR “acute kidney failure*” OR “acute renal failure*” OR “chemically-induced liver toxicit*” OR “chemically induced liver toxicit*” OR “drug-induced acute liver injur*” OR “drug induced acute liver injur*” OR “toxic hepatitis” OR “toxic hepatitides” OR “drug-induced liver disease*” OR “drug induced liver disease*” OR “drug-induced liver injur*” OR “drug induced liver injur*” OR “drug induced hepatitides” OR “drug-induced hepatitides” OR “drug-induced hepatitis” OR “drug induced hepatitis” OR “tendon injur*” ) | 7672 |
|  | #3 | ab(“older adult*” OR “over 65” OR “elderly” OR “over 80” OR aged OR elderly) OR ti(“older adult*” OR “over 65” OR “elderly” OR “over 80” OR aged OR elderly) | 352549 |
|  | **#4** | **#1 AND #2 AND #3** | **6** |
| **PsycINFO** | #1 | Title: “Anti-Bacterial agent*” OR Aminoglycoside* OR “beta-lactam*” OR Penicillin* OR Monobactam* OR Carbapenem* OR Cephalosporin* OR Fluoroquinolone* OR Glycopeptide* OR lipoglycopeptide* OR Macrolide* OR Oxazolidinone* OR Rifamycin* OR Sulfonamide* OR Streptogramin* OR Tetracycline* OR lincosamide* OR chloramphenicol OR daptomycin OR Fosfomycin OR metronidazole OR mupirocin OR nitrofurantoin OR Abstract: “Anti-Bacterial agent*” OR Aminoglycoside* OR “beta-lactam*” OR Penicillin* OR Monobactam* OR Carbapenem* OR Cephalosporin* OR Fluoroquinolone* OR Glycopeptide* OR lipoglycopeptide* OR Macrolide* OR Oxazolidinone* OR Rifamycin* OR Sulfonamide* OR Streptogramin* OR Tetracycline* OR lincosamide* OR chloramphenicol OR daptomycin OR Fosfomycin OR metronidazole OR mupirocin OR nitrofurantoin | 19337 |
|  | #2 | Title: “acute kidney injur*” OR “acute renal injur*” OR “acute renal insufficienc*” OR “acute kidney insufficienc*” OR “acute kidney failure*” OR “acute renal failure*” OR “chemically-induced liver toxicit*” OR “chemically induced liver toxicit*” OR “drug-induced acute liver injur*” OR “drug induced acute liver injur*” OR “toxic hepatitis” OR “toxic hepatitides” OR “drug-induced liver disease*” OR “drug induced liver disease*” OR “drug-induced liver injur*” OR “drug induced liver injur*” OR “drug induced hepatitides” OR “drug-induced hepatitides” OR “drug-induced hepatitis” OR “drug induced hepatitis” OR “tendon injur*” OR Abstract: “acute kidney injur*” OR “acute renal injur*” OR “acute renal insufficienc*” OR “acute kidney insufficienc*” OR “acute kidney failure*” OR “acute renal failure*” OR “chemically-induced liver toxicit*” OR “chemically induced liver toxicit*” OR “drug-induced acute liver injur*” OR “drug induced acute liver injur*” OR “toxic hepatitis” OR “toxic hepatitides” OR “drug-induced liver disease*” OR “drug induced liver disease*” OR “drug-induced liver injur*” OR “drug induced liver injur*” OR “drug induced hepatitides” OR “drug-induced hepatitides” OR “drug-induced hepatitis” OR “drug induced hepatitis” OR “tendon injur*” | 341 |
|  | #3 | Title: “older adult*” OR “over 65” OR “elderly” OR “over 80” OR aged OR elderly OR Abstract: “older adult*” OR “over 65” OR “elderly” OR “over 80” OR aged OR elderly | 362183 |
|  | **#4** | **#1 AND #2 AND #3** | **1** |
