# Supplementary material for: Risk of antimicrobial-associated organ injury among the older adults: a systematic review and meta-analysis
Source: BMC Geriatr. 2021 Nov 1;21:617. doi: 10.1186/s12877-021-02512-3 (PMC8561875; doi:10.1186/s12877-021-02512-3)
Supplement: Supplementary file 3 — Additional file 3. Study appraisals – Risk of Bias assessment using Newcastle-Ottawa scale. [file 12877_2021_2512_MOESM3_ESM.docx]

**Study appraisals – Risk of Bias assessment using Newcastle-Ottawa scale**

**MODIFIED NEWCASTLE - OTTAWA QUALITY ASSESSMENT SCALE: COHORT STUDIES**

**EndNote number:** #1188 **Author:** Ahmed et al. 2018 **Title:** Risk of adverse outcomes following urinary tract infection in older people with renal impairment: Retrospective cohort study using linked health record data

Note: A study can be awarded a maximum of one star for each numbered item within the Selection and Outcome categories. A maximum of two stars can be given for Comparability

| **Criterion and Decision rules** | **Score (Star*=1 point. Two stars**=2points. No star= 0 point)** | **Location in text** |
| --- | --- | --- |
| **SELECTION** |  |  |
| 1. *Representativeness of the exposed cohort*    1. Truly representative of the elderly population (at least 65 years old) *****    2. Subgroup of the exposed population are elderly (at least 65 years) *****    3. Exposed population was under 65 years    4. No description of the derivation of the exposed cohort | **1** | **Design and participants:** “Patients were eligible in the study if … they were > or = 65 years old …” |
| 1. *Selection of the non-exposed cohort*    1. Drawn from the same community as the exposed cohort *****    2. Drawn from a different source    3. No description of the derivation of the non-exposed cohort | **1** | **Design and participants:** Selected from the same population as exposed group |
| 1. *Ascertainment of exposure*    1. Secure record (i.e. medical records) describing initial and/or ongoing exposure to antimicrobial *****    2. Self-reporting of exposure    3. No description of exposure | **1** | **Design and participants:** “… a prescription code indicating same-day prescribing of empirical antibiotic …” |
| 1. *Demonstration of the absence of the outcome of interest at the beginning of the study*    1. Yes *****    2. No | **1** | **Exposures:** “… used the most recent creatinine value … and data for patient age, gender, and ethnicity to calculate eGFR as per Modification of Diet in Renal Disease (MDRD) protocol.” |
| **COMPARABILITY** |  |  |
| 1. *Comparability of cohorts based on design and analysis*    1. Study controls for sex, age, and disease severity at baseline *    2. Study controls for the above plus any other additional factors ******    3. Limited or no attempt to control for differences between the cohorts | **2** | **Statistical analyses:** “…adjusted for potential confounders … including age, index of multiple deprivation score quintile, Charlson’s co-morbidity score, … diabetes, dementia, coronary heart disease, stroke, cancer, heart failure and poly pharmacy.” |
| **OUTCOME** |  |  |
| 1. *Assessment of Outcome*     1. Independent or blind assessment, or confirmation of the outcome by reference to secure records (i.e. medical records, laboratory results etc) *****    2. Identified through ICD codes on database records *****    3. Self-report i.e. no reference to original medical records to confirm the outcome    4. No description | **1** | **Outcomes: “…**assessed the impact of our stated exposures on the following outcomes for patients: … hospitalisation for UTI, sepsis or acute kidney injury (AKI) within 14 days following the incident UTI and ascertained from ICD-10 codes …” |
| 1. *Was follow-up long enough for outcomes to occur*    1. Yes *****    2. No    3. Length of follow-up not stated | **1** | **Outcomes:** “… assessed the impact of our stated exposures on the following outcomes for patients, … or acute kidney injury (AKI) within 14 days following the incident UTI ascertained from ICD-10 codes …” |
| 1. *Adequacy of follow-up of cohorts*    1. Complete follow-up – all subjects accounted for *****    2. Loss to follow-up less than 20% or description given for those lost *****    3. Follow-up rate less than 50% and no description of those lost    4. No statement | **1** | **Outcomes:** Data analysed retrospectively |
| **TOTAL SCORE** | **9** |  |

**MODIFIED NEWCASTLE - OTTAWA QUALITY ASSESSMENT SCALE: COHORT STUDIES**

**EndNote number:** #1187 **Author:** Ahmed et al., 2019 **Title:** Choice of empirical antibiotic therapy and adverse outcomes in older adults with suspected urinary tract infection: Cohort study

Note: A study can be awarded a maximum of one star for each numbered item within the Selection and Outcome categories. A maximum of two stars can be given for Comparability

| **Criterion and Decision rules** | **Score (Star*=1 point. Two stars**=2points. No star= 0 point)** | **Location in text** |
| --- | --- | --- |
| **SELECTION** |  |  |
| 1. *Representativeness of the exposed cohort*    1. Truly representative of the elderly population (at least 65 years old) *****    2. Subgroup of the exposed population are elderly (at least 65 years) *****    3. Exposed population was under 65 years    4. No description of the derivation of the exposed cohort | **1** | **Design and participants: “**Patients were eligible for inclusion if … they were > or = 65 years old, …” |
| 1. *Selection of the non-exposed cohort*    1. Drawn from the same community as the exposed cohort *****    2. Drawn from a different source    3. No description of the derivation of the non-exposed cohort | **1** | **Design and participants:** Drawn from the same cohort |
| 1. *Ascertainment of exposure*    1. Secure record (i.e. medical records) describing initial and/or ongoing exposure to antimicrobial *****    2. Self-reporting of exposure    3. No description of exposure | **1** | **Design and participants:** “We identified eligible patients with a Read code indicating an incident primary care presentation with a suspected UTI … and a same day prescription code indicating empirical prescribing of a relevant antibiotic.” |
| 1. *Demonstration of the absence of the outcome of interest at the beginning of the study*    1. Yes *****    2. No | **0** | No demonstration of absence of outcome of interest prior to antibiotic exposure. |
| **COMPARABILITY** |  |  |
| 1. *Comparability of cohorts based on design and analysis*    1. Study controls for sex, age, and disease severity at baseline *    2. Study controls for the above plus any other additional factors ******    3. Limited or no attempt to control for differences between the cohorts | **2** | **Statistical analyses:** “…variables included in the logistic regression model were age, sex, dementia, liver disease, cancer, rheumatoid arthritis, urinary incontinence, eGFR, polypharmacy, coronary heart disease, renal disease, stroke, respiratory disease, type 2 diabetes mellitus …” |
| **OUTCOME** |  |  |
| 1. *Assessment of Outcome*     1. Independent or blind assessment, or confirmation of the outcome by reference to secure records (i.e. medical records, laboratory results etc) *****    2. Identified through ICD codes on database records *****    3. Self-report i.e. no reference to original medical records to confirm the outcome    4. No description | **1** | **Outcomes:** “… AKI within 14 days after incident UTI, ascertained by from ICD-10 codes recorded in linked hospital admission data …” |
| 1. *Was follow-up long enough for outcomes to occur*    1. Yes *****    2. No    3. Length of follow-up not stated | **1** | **Outcomes:** “…hospitalisation for …, or AKI within 14 days following incident UTI …” |
| 1. *Adequacy of follow-up of cohorts*    1. Complete follow-up – all subjects accounted for *****    2. Loss to follow-up less than 20% or description given for those lost *****    3. Follow-up rate less than 50% and no description of those lost    4. No statement | **1** | **Results:** Retrospective analysis therefore no loss to follow up |
| **TOTAL SCORE** | **8** |  |

**MODIFIED NEWCASTLE - OTTAWA QUALITY ASSESSMENT SCALE: COHORT STUDIES**

**EndNote number:** #1186 **Author:** Ahmed et al., 2016 **Title:** The effectiveness and safety of two prophylactic antibiotic regimes

Note: A study can be awarded a maximum of one star for each numbered item within the Selection and Outcome categories. A maximum of two stars can be given for Comparability

| **Criterion and Decision rules** | **Score (Star*=1 point. Two stars**=2points. No star= 0 point)** | **Location in text** |
| --- | --- | --- |
| **SELECTION** |  |  |
| 1. *Representativeness of the exposed cohort*    1. Truly representative of the elderly population (at least 65 years old) *****    2. Subgroup of the exposed population are elderly (at least 65 years) *****    3. Exposed population was under 65 years    4. No description of the derivation of the exposed cohort | **1** | **Patients, methods, and definitions:** “… prospective cohort of hip-fracture patients who received antibiotic prophylaxis…”  **Results:** Mean age of exposed cohort was 81.6 years (table 2). |
| 1. *Selection of the non-exposed cohort*    1. Drawn from the same community as the exposed cohort *****    2. Drawn from a different source    3. No description of the derivation of the non-exposed cohort | **1** | **Patients, methods, and definitions:** “This study compares retrospective consecutive case series with a prospective cohort group of hip-fractured patients who received antibiotic prophylaxis…”  **Results:** Mean age of control group was 81 years (table 2) |
| 1. *Ascertainment of exposure*    1. Secure record (i.e. medical records) describing initial and/or ongoing exposure to antimicrobial *****    2. Self-reporting of exposure    3. No description of exposure | **1** | **Patients, methods, and definitions:** “Group I patients (control group) were administered 1500mg cefuroxime intravenously at induction of anaesthesia … whereas group II patients (exposed group) received 1 gram of flucloxacillin plus gentamicin 5 mg/kg at the induction of anaesthesia …” |
| 1. *Demonstration of the absence of the outcome of interest at the beginning of the study*    1. Yes *****    2. No | **1** | **Patients, methods, and definitions:** “To analyse AKI in detail, on the basis of pre-operative eGFR we classified patients into three subgroups i.e. normal preoperative renal function, mild or moderate preoperative renal dysfunction…” |
| **COMPARABILITY** |  |  |
| 1. *Comparability of cohorts based on design and analysis*    1. Study controls for sex, age, and disease severity at baseline *    2. Study controls for the above plus any other additional factors ******    3. Limited or no attempt to control for differences between the cohorts | **2** | **Statistical analysis:** “Logistic regression analysis was performed to control confounding factors i.e. age gender, ASA grade, surgeon grade and type of anaesthesia.” |
| **OUTCOME** |  |  |
| 1. *Assessment of Outcome*     1. Independent or blind assessment, or confirmation of the outcome by reference to secure records (i.e. medical records, laboratory results etc) *****    2. Identified through ICD codes on database records *****    3. Self-report i.e. no reference to original medical records to confirm the outcome    4. No description | **1** | **Patients, methods, and definitions:** “AKI is defined as an increase in serum creatinine of >= 26.4 umol/l, or > = 150 -200%, from baseline. We applied AKIN categories to grade severity of AKI.” |
| 1. *Was follow-up long enough for outcomes to occur*    1. Yes *****    2. No    3. Length of follow-up not stated | **0** | Not clearly stated in units of time e.g. days. Only stated as “post operatively” and “at discharge” |
| 1. *Adequacy of follow-up of cohorts*    1. Complete follow-up – all subjects accounted for *****    2. Loss to follow-up less than 20% or description given for those lost *****    3. Follow-up rate less than 50% and no description of those lost    4. No statement | **1** | **Results:** “… 744 patients were in group I (cefuroxime) and 756 were in group II (flucloxacillin + gentamicin)” |
| **TOTAL SCORE** | **8** |  |

**MODIFIED NEWCASTLE - OTTAWA QUALITY ASSESSMENT SCALE: COHORT STUDIES**

**EndNote number**: #1115 **Author:** Baciewicz etal., 2003 **Title:** Aminoglycoside-associated nephrotoxicity in the elderly

Note: A study can be awarded a maximum of one star for each numbered item within the Selection and Outcome categories. A maximum of two stars can be given for Comparability

| **Criterion and Decision rules** | **Score (Star*=1 point. Two stars**=2points. No star= 0 point)** | **Location in text** |
| --- | --- | --- |
| **SELECTION** |  |  |
| 1. *Representativeness of the exposed cohort*    1. Truly representative of the elderly population (at least 65 years old) *****    2. Subgroup of the exposed population are elderly (at least 65 years) *****    3. Exposed population was under 65 years    4. No description of the derivation of the exposed cohort | **1** | **Methods:** “This prospective observational audit evaluated the physician prescribing of SD and MD aminoglycosides in elderly patients (>=65 years) …” |
| 1. *Selection of the non-exposed cohort*    1. Drawn from the same community as the exposed cohort *****    2. Drawn from a different source    3. No description of the derivation of the non-exposed cohort | **1** | **Methods:** “The audit group consisted of patients who received SD aminoglycosides while the control group received MD aminoglycosides.” |
| 1. *Ascertainment of exposure*    1. Secure record (i.e. medical records) describing initial and/or ongoing exposure to antimicrobial *****    2. Self-reporting of exposure    3. No description of exposure | **1** | **Methods:** “Gentamicin and tobramycin were the two aminoglycosides most prescribed. Elderly patients with physician prescription for either agent was identified through the hospital-wide computer system …” |
| 1. *Demonstration of the absence of the outcome of interest at the beginning of the study*    1. Yes *****    2. No | **1** | **Methods**: “Nephrotoxicity was defined as sustained increase (for at least 2 days) of serum creatinine of 0.5 mg/dl from that determined prior to initiating aminoglycoside therapy” |
| **COMPARABILITY** |  |  |
| 1. *Comparability of cohorts based on design and analysis*    1. Study controls for sex, age, and disease severity at baseline *    2. Study controls for the above plus any other additional factors ******    3. Limited or no attempt to control for differences between the cohorts | **0** | **Methods:** Although the study authors stated that “regression analysis was performed to determine whether any factor(s) significantly influenced nephrotoxicity associated with SD or MD aminoglycoside therapy”, there was no attempt to specify possible confounders. |
| **OUTCOME** |  |  |
| 1. *Assessment of Outcome*     1. Independent or blind assessment, or confirmation of the outcome by reference to secure records (i.e. medical records, laboratory results etc) *****    2. Identified through ICD codes on database records *****    3. Self-report i.e. no reference to original medical records to confirm the outcome    4. No description | **1** | **Methods**: “Nephrotoxicity was defined as sustained increase (for at least 2 days) of serum creatinine of 0.5 mg/dl from that determined prior to initiating aminoglycoside therapy” |
| 1. *Was follow-up long enough for outcomes to occur*    1. Yes *****    2. No    3. Length of follow-up not stated | **0** | Follow up length not stated |
| 1. *Adequacy of follow-up of cohorts*    1. Complete follow-up – all subjects accounted for *****    2. Loss to follow-up less than 20% or description given for those lost *****    3. Follow-up rate less than 50% and no description of those lost    4. No statement | **1** | **Results:** No loss to follow up reported and all recruited participants accounted for in the results. |
| **TOTAL SCORE** | **6** |  |

**MODIFIED NEWCASTLE - OTTAWA QUALITY ASSESSMENT SCALE: COHORT STUDIES**

**EndNote number:** #1059 **Author:** Bright-Thomas et al., 2016**. Title:** Drug-related hepatitis in patients treated with standard antituberculosis chemotherapy over a 30-year period

Note: A study can be awarded a maximum of one star for each numbered item within the Selection and Outcome categories. A maximum of two stars can be given for Comparability

| **Criterion and Decision rules** | **Score (Star*=1 point. Two stars**=2points. No star= 0 point)** | **Location in text** |
| --- | --- | --- |
| **SELECTION** |  |  |
| 1. *Representativeness of the exposed cohort*    1. Truly representative of the elderly population (at least 65 years old) *****    2. Subgroup of the exposed population are elderly (at least 65 years) *****    3. Exposed population was under 65 years    4. No description of the derivation of the exposed cohort | **1** | **Methods: “**Data on all patients treated for active TB at any site were examined over a 30-year period commencing 1 January 1981 …”  **Results:** Rate of hepatitis among the exposed by age presented in table 3. |
| 1. *Selection of the non-exposed cohort*    1. Drawn from the same community as the exposed cohort *****    2. Drawn from a different source    3. No description of the derivation of the non-exposed cohort | **1** | Drawn from same cohort as the exposed. |
| 1. *Ascertainment of exposure*    1. Secure record (i.e. medical records) describing initial and/or ongoing exposure to antimicrobial *****    2. Self-reporting of exposure    3. No description of exposure | **1** | **Methods:** “All patients were treated with 6 months of rifampicin (R, RMP) and isoniazid (H, INH), with 2 months of initial pyrazinamide (Z, PZA) and ethambutol (E, EMB), …” |
| 1. *Demonstration of the absence of the outcome of interest at the beginning of the study*    1. Yes *****    2. No | **0** | No evidence of baseline measurements to determine absence of hepatitis among the participants. |
| **COMPARABILITY** |  |  |
| 1. *Comparability of cohorts based on design and analysis*    1. Study controls for sex, age, and disease severity at baseline *    2. Study controls for the above plus any other additional factors ******    3. Limited or no attempt to control for differences between the cohorts | **1** | **Methods:** “… logistic regressions were used to examine the effect of ethnic origin, age and sex on the rate of drug-induced hepatitis.” |
| **OUTCOME** |  |  |
| 1. *Assessment of Outcome*     1. Independent or blind assessment, or confirmation of the outcome by reference to secure records (i.e. medical records, laboratory results etc) *****    2. Identified through ICD codes on database records *****    3. Self-report i.e. no reference to original medical records to confirm the outcome    4. No description | **1** | **Methods:** “… the diagnosis of hepatotoxicity was liver transaminases (aspartate transaminase/alanine transaminase) rising to >5 times the upper limit of normal, or a rising bilirubin level.” |
| 1. *Was follow-up long enough for outcomes to occur*    1. Yes *****    2. No    3. Length of follow-up not stated | **1** | **Methods:** “All patients were treated with 6 months of rifampicin (R, RMP) and isoniazid (H, INH), with 2 months of initial pyrazinamide (Z, PZA) and ethambutol (E, EMB), with treatment prolonged to 12 months in those patients with meningeal involvement …” |
| 1. *Adequacy of follow-up of cohorts*    1. Complete follow-up – all subjects accounted for *****    2. Loss to follow-up less than 20% or description given for those lost *****    3. Follow-up rate less than 50% and no description of those lost    4. No statement | **1** | **Results:** No evidence of loss to follow up. |
| **TOTAL SCORE** | **7** |  |

**MODIFIED NEWCASTLE - OTTAWA QUALITY ASSESSMENT SCALE: COHORT STUDIES**

**EndNote number**: #1030.  **Author:** Carreno et al., 2013 **Title:** Comparative Incidence of Nephrotoxicity by Age Group among Adult Patients Receiving Vancomycin

Note: A study can be awarded a maximum of one star for each numbered item within the Selection and Outcome categories. A maximum of two stars can be given for Comparability

| **Criterion and Decision rules** | **Score (Star*=1 point. Two stars**=2points. No star= 0 point)** | **Location in text** |
| --- | --- | --- |
| **SELECTION** |  |  |
| 1. *Representativeness of the exposed cohort*    1. Truly representative of the elderly population (at least 65 years old) *****    2. Subgroup of the exposed population are elderly (at least 65 years) *****    3. Exposed population was under 65 years    4. No description of the derivation of the exposed cohort | **1** | **Study design:** “Patients included were aged at least 18 years, … Patients were categorized by age as young (18–64 years), older adults (65–79 years) and very elderly (C80 years).” |
| 1. *Selection of the non-exposed cohort*    1. Drawn from the same community as the exposed cohort *****    2. Drawn from a different source    3. No description of the derivation of the non-exposed cohort | **1** | Selected from the same cohort as the exposed group. |
| 1. *Ascertainment of exposure*    1. Secure record (i.e. medical records) describing initial and/or ongoing exposure to antimicrobial *****    2. Self-reporting of exposure    3. No description of exposure | **1** | **Study design:** “Patients … received at least four consecutive vancomycin doses, and had at least one recorded vancomycin serum trough concentration during therapy …” |
| 1. *Demonstration of the absence of the outcome of interest at the beginning of the study*    1. Yes *****    2. No | **1** | **Study design:** “Patients were excluded if they had concurrent acute kidney injury prior to initiation of vancomycin (defined as an increase in serum creatinine of 0.3 mg/dL or 50% within 48 h prior to starting vancomycin, or if urine output was <0.5 mL/kg/h for at least 6 h immediately before the initiation of vancomycin)” |
| **COMPARABILITY** |  |  |
| 1. *Comparability of cohorts based on design and analysis*    1. Study controls for sex, age, and disease severity at baseline *    2. Study controls for the above plus any other additional factors ******    3. Limited or no attempt to control for differences between the cohorts | **1** | **Statistical analysis:** “… a multivariable logistic regression model was constructed to determine the association between the age group and nephrotoxicity and acute kidney injury. Age was entered into the model and any variables found to have an association with the outcome of interest (p<0.20) …” |
| **OUTCOME** |  |  |
| 1. *Assessment of Outcome*     1. Independent or blind assessment, or confirmation of the outcome by reference to secure records (i.e. medical records, laboratory results etc) *****    2. Identified through ICD codes on database records *****    3. Self-report i.e. no reference to original medical records to confirm the outcome    4. No description | **1** | **Study design**: “The primary outcome of interest was nephrotoxicity, defined as an abrupt (within 48 h) increase in serum creatinine of 0.5 mg/dL or 50% above baseline for at least two consecutive measurements**”** |
| 1. *Was follow-up long enough for outcomes to occur*    1. Yes *****    2. No    3. Length of follow-up not stated | **0** | **Study design:** Authors did not clearly state how long the patients were monitored for the outcome following exposure. |
| 1. *Adequacy of follow-up of cohorts*    1. Complete follow-up – all subjects accounted for *****    2. Loss to follow-up less than 20% or description given for those lost *****    3. Follow-up rate less than 50% and no description of those lost    4. No statement | **1** | No loss of participants during follow up since it was a retrospective study. |
| **TOTAL SCORE** | **7** |  |

**MODIFIED NEWCASTLE - OTTAWA QUALITY ASSESSMENT SCALE: COHORT STUDIES**

**EndNote number:** #950 **Author:** Craig et al., 2012 **Title:** Is prophylactic Gentamicin associated with acute kidney injury in patients undergoing surgery for fractured neck of femur?

Note: A study can be awarded a maximum of one star for each numbered item within the Selection and Outcome categories. A maximum of two stars can be given for Comparability

| **Criterion and Decision rules** | **Score (Star*=1 point. Two stars**=2points. No star= 0 point)** | **Location in text** |
| --- | --- | --- |
| **SELECTION** |  |  |
| 1. *Representativeness of the exposed cohort*    1. Truly representative of the elderly population (at least 65 years old) *****    2. Subgroup of the exposed population are elderly (at least 65 years) *****    3. Exposed population was under 65 years    4. No description of the derivation of the exposed cohort | **1** | **Methods:** “Administration of single dose prophylactic Gentamicin was the exposure and the development of AKI was the outcome measure. Between September and November 2010 data was recorded from 100 consecutive patients admitted to our unit who underwent surgery for fractured neck of femur.” **Results:** “…the average age of the study group was 80.3 years.” |
| 1. *Selection of the non-exposed cohort*    1. Drawn from the same community as the exposed cohort *****    2. Drawn from a different source    3. No description of the derivation of the non-exposed cohort | **1** | Methods: “Case controls were identified from the departmental data base between 2003 and 2004 before the inclusion of Gentamicin into our prophylactic regimen. These individuals all received three intravenous doses of 1.5 g of Cefuroxime (one at induction and the following two in the immediate 16 h postoperative period).” **Results:** The average age of the control group was 83.6 years.” |
| 1. *Ascertainment of exposure*    1. Secure record (i.e. medical records) describing initial and/or ongoing exposure to antimicrobial *****    2. Self-reporting of exposure    3. No description of exposure | **1** | **Methods:** “All patients received antibiotic prophylaxis in accordance with the departmental protocol: Co-Amoxiclav 1.5 g and Gentamicin 240 mg given intravenously at induction.” |
| 1. *Demonstration of the absence of the outcome of interest at the beginning of the study*    1. Yes *****    2. No | **1** | **Methods**: “Preoperative creatinine levels … measured within 24 hours of surgery.” |
| **COMPARABILITY** |  |  |
| 1. *Comparability of cohorts based on design and analysis*    1. Study controls for sex, age, and disease severity at baseline *    2. Study controls for the above plus any other additional factors ******    3. Limited or no attempt to control for differences between the cohorts | **1** | **Methods:** “Individuals were selected at random and matched according to age (2 years), sex and operative procedure.” |
| **OUTCOME** |  |  |
| 1. *Assessment of Outcome*     1. Independent or blind assessment, or confirmation of the outcome by reference to secure records (i.e. medical records, laboratory results etc) *****    2. Identified through ICD codes on database records *****    3. Self-report i.e. no reference to original medical records to confirm the outcome    4. No description | **1** | **Methods:** “Acute kidney injury (AKI) was defined as a ‘rise in serum creatinine level of greater than 50% of the baseline’’ |
| 1. *Was follow-up long enough for outcomes to occur*    1. Yes *****    2. No    3. Length of follow-up not stated | **1** | **Methods**: “Data was recorded as follows; … preoperative creatinine levels, and daily post-operative creatinine levels for up to 7 days.” |
| 1. *Adequacy of follow-up of cohorts*    1. Complete follow-up – all subjects accounted for *****    2. Loss to follow-up less than 20% or description given for those lost *****    3. Follow-up rate less than 50% and no description of those lost    4. No statement | **1** | **Results:** No loss during follow up. All subjects accounted for. |
| **TOTAL SCORE** | **8** |  |

**MODIFIED NEWCASTLE - OTTAWA QUALITY ASSESSMENT SCALE: COHORT STUDIES**

**EndNote number:** #946 **Author:** Crellin et al., 2018 **Title:** Trimethoprim use for urinary tract infection and risk of adverse outcomes in older patients: Cohort study

Note: A study can be awarded a maximum of one star for each numbered item within the Selection and Outcome categories. A maximum of two stars can be given for Comparability

| **Criterion and Decision rules** | **Score (Star*=1 point. Two stars**=2points. No star= 0 point)** | **Location in text** |
| --- | --- | --- |
| **SELECTION** |  |  |
| 1. *Representativeness of the exposed cohort*    1. Truly representative of the elderly population (at least 65 years old) *****    2. Subgroup of the exposed population are elderly (at least 65 years) *****    3. Exposed population was under 65 years    4. No description of the derivation of the exposed cohort | **1** | **Participants, exposures, and outcomes:** “We identified all adults aged 65 years and over during the study period (April 1997 to September 2015).” |
| 1. *Selection of the non-exposed cohort*    1. Drawn from the same community as the exposed cohort *****    2. Drawn from a different source    3. No description of the derivation of the non-exposed cohort | **1** | Drawn from the same community as the exposed. |
| 1. *Ascertainment of exposure*    1. Secure record (i.e. medical records) describing initial and/or ongoing exposure to antimicrobial *****    2. Self-reporting of exposure    3. No description of exposure | **1** | **Participants, exposures, and outcomes:** “The date of inclusion was the day of initiation for any of five antibiotic drugs (trimethoprim, amoxicillin, cefalexin, ciprofloxacin, and nitrofurantoin) recorded up to three days after a primary care morbidity code for uncomplicated UTI.” |
| 1. *Demonstration of the absence of the outcome of interest at the beginning of the study*    1. Yes *****    2. No | **1** | **Covariates: “**Baseline renal function was defined using the most recent biochemical test results recorded in primary care at any time before each UTI treated with antibiotics. We used serum creatinine test results to calculate estimated glomerular filtration rate using the Chronic Kidney Disease Epidemiology Collaboration (CKDEPI) equation.” |
| **COMPARABILITY** |  |  |
| 1. *Comparability of cohorts based on design and analysis*    1. Study controls for sex, age, and disease severity at baseline *    2. Study controls for the above plus any other additional factors ******    3. Limited or no attempt to control for differences between the cohorts | **2** | **Statistical analysis:** “We initially adjusted for sex and age only, and then fitted an adjusted model using sex, age, calendar period, chronic comorbidities, baseline renal function, history of renal or urological disease, and use of renin-angiotensin system blockers and potassium-sparing diuretics.” |
| **OUTCOME** |  |  |
| 1. *Assessment of Outcome*     1. Independent or blind assessment, or confirmation of the outcome by reference to secure records (i.e. medical records, laboratory results etc) *****    2. Identified through ICD codes on database records *****    3. Self-report i.e. no reference to original medical records to confirm the outcome    4. No description | **1** | **Outcomes:** “Acute kidney injury was defined as hospital admission with acute kidney injury using ICD-10 (international classification of diseases, 10th revision) codes recorded in any diagnostic position of any inpatient episode starting within 14 days of antibiotic initiation.” |
| 1. *Was follow-up long enough for outcomes to occur*    1. Yes *****    2. No    3. Length of follow-up not stated | **1** | **Outcomes:** “Acute kidney injury was defined as hospital admission with acute kidney injury using ICD-10 codes recorded in any diagnostic position of any inpatient episode starting within 14 days of antibiotic initiation.” |
| 1. *Adequacy of follow-up of cohorts*    1. Complete follow-up – all subjects accounted for *****    2. Loss to follow-up less than 20% or description given for those lost *****    3. Follow-up rate less than 50% and no description of those lost    4. No statement | **1** | No loss to follow up since it was a retrospective cohort study. |
| **TOTAL SCORE** | **9** |  |

**MODIFIED NEWCASTLE - OTTAWA QUALITY ASSESSMENT SCALE: COHORT STUDIES**

**EndNote number:** #826 **Author:** Fraisse et al., 2014 **Title:** Aminoglycosides use in patients over 75 years old

Note: A study can be awarded a maximum of one star for each numbered item within the Selection and Outcome categories. A maximum of two stars can be given for Comparability

| **Criterion and Decision rules** | **Score (Star*=1 point. Two stars**=2points. No star= 0 point)** | **Location in text** |
| --- | --- | --- |
| **SELECTION** |  |  |
| 1. *Representativeness of the exposed cohort*    1. Truly representative of the elderly population (at least 65 years old) *****    2. Subgroup of the exposed population are elderly (at least 65 years) *****    3. Exposed population was under 65 years    4. No description of the derivation of the exposed cohort | **1** | **Methods**: “The inclusion criteria were patients 75 years and older hospitalised for >24 h who received at least one dose of aminoglycoside, after March 2011 …” |
| 1. *Selection of the non-exposed cohort*    1. Drawn from the same community as the exposed cohort *****    2. Drawn from a different source    3. No description of the derivation of the non-exposed cohort | **1** | Drawn from the same cohort as the exposed |
| 1. *Ascertainment of exposure*    1. Secure record (i.e. medical records) describing initial and/or ongoing exposure to antimicrobial *    2. Self-reporting of exposure    3. No description of exposure | **1** | **Methods:** “The inclusion criteria were patients 75 years and older hospitalised for >24 h who received at least one dose of aminoglycoside” |
| 1. *Demonstration of the absence of the outcome of interest at the beginning of the study*    1. Yes *****    2. No | **1** | **Methods:** “A standardised data collection sheet were retrospectively filled in from the medical charts including: demographics (sex, age), weight, serum creatinine and glomerular filtration rate (GFR) estimated by the Modification of Diet in Renal Disease (MDRD) formula” |
| **COMPARABILITY** |  |  |
| 1. *Comparability of cohorts based on design and analysis*    1. Study controls for sex, age, and disease severity at baseline *    2. Study controls for the above plus any other additional factors ******    3. Limited or no attempt to control for differences between the cohorts | **2** | **Statistical analysis:** “Multivariate logistic regression analysis was carried out for the factors associated with the risk of renal failure by the univariate analysis (P < 0.20) …”  **Table 3**: Factors with p < 0.20 were sex, nephrotoxic drugs, prescription duration, number of administrations per day, and monitoring of residual level |
| **OUTCOME** |  |  |
| 1. *Assessment of Outcome*     1. Independent or blind assessment, or confirmation of the outcome by reference to secure records (i.e. medical records, laboratory results etc) *****    2. Identified through ICD codes on database records *****    3. Self-report i.e. no reference to original medical records to confirm the outcome    4. No description | **1** | **Methods:** “Nephrotoxicity was defined by an increase of creatinine level over 25% between admission and the last available measure was used to define nephrotoxicity.” |
| 1. *Was follow-up long enough for outcomes to occur*    1. Yes *****    2. No    3. Length of follow-up not stated | **0** | Length of follow up time not stated in methods**.** |
| 1. *Adequacy of follow-up of cohorts*    1. Complete follow-up – all subjects accounted for *****    2. Loss to follow-up less than 20% or description given for those lost *****    3. Follow-up rate less than 50% and no description of those lost    4. No statement | **1** | **Results:** All included subjects accounted for. |
| **TOTAL SCORE** | **8** |  |

**MODIFIED NEWCASTLE - OTTAWA QUALITY ASSESSMENT SCALE: COHORT STUDIES**

**EndNote number:** #816 **Author:** Gandhi et al., 2010 **Title:** Calcium-channel blocker-clarithromycin drug interactions and acute kidney injury

Note: A study can be awarded a maximum of one star for each numbered item within the Selection and Outcome categories. A maximum of two stars can be given for Comparability

| **Criterion and Decision rules** | **Score (Star*=1 point. Two stars**=2points. No star= 0 point)** | **Location in text** |
| --- | --- | --- |
| **SELECTION** |  |  |
| 1. *Representativeness of the exposed cohort*    1. Truly representative of the elderly population (at least 65 years old) *****    2. Subgroup of the exposed population are elderly (at least 65 years) *****    3. Exposed population was under 65 years    4. No description of the derivation of the exposed cohort | **1** | **Data sources:** “We used the Ontario Drug Benefit Program database to identify prescription drug use. This database contains highly accurate records of all outpatient prescriptions dispensed to patients aged 65 years or older, with an error rate of less than 1%.” |
| 1. *Selection of the non-exposed cohort*    1. Drawn from the same community as the exposed cohort *****    2. Drawn from a different source    3. No description of the derivation of the non-exposed cohort | **1** | Drawn from same cohort |
| 1. *Ascertainment of exposure*    1. Secure record (i.e. medical records) describing initial and/or ongoing exposure to antimicrobial *****    2. Self-reporting of exposure    3. No description of exposure | **1** | **Data Sources: “**We also used the ICES Physician Database to ascertain antibiotic prescriber information”  **Patients:** “The date of the clarithromycin or azithromycin prescription served as the index date (referred to as cohort entry date or start time for follow-up)” |
| 1. *Demonstration of the absence of the outcome of interest at the beginning of the study*    1. Yes *****    2. No | **1** | **Data sources:** “International Classification of Diseases, 9th revision (ICD-9; pre-2002) and 10th revision (ICD-10; post-2002) codes were used to assess baseline comorbidities in the 5 years prior to receipt of the relevant co-prescription”  **Patients:** “We excluded the following patients, … those with a history of end-stage renal disease receiving chronic dialysis because the assessment of acute kidney injury is no longer relevant in such individuals.” |
| **COMPARABILITY** |  |  |
| 1. *Comparability of cohorts based on design and analysis*    1. Study controls for sex, age, and disease severity at baseline *    2. Study controls for the above plus any other additional factors ******    3. Limited or no attempt to control for differences between the cohorts | **2** | **Statistical analysis: “**We adjusted for 17 potential confounders: age, sex, baseline use of angiotensin-converting enzyme inhibitors or angiotensin receptor blockers, nonsteroidal anti-inflammatory agents, oral hypoglycaemic agents or insulin, non-potassium-sparing diuretics or potassium-sparing diuretics, statins, β-blockers, β2- agonists, anticholinergics, and corticosteroids (with all drugs defined by evidence of at least 1 prescription in the preceding 6 months), as well as baseline evidence of chronic kidney disease, coronary artery disease, cerebrovascular disease, peripheral vascular disease, congestive heart failure, and major cancers …” |
| **OUTCOME** |  |  |
| 1. *Assessment of Outcome*     1. Independent or blind assessment, or confirmation of the outcome by reference to secure records (i.e. medical records, laboratory results etc) *****    2. Identified through ICD codes on database records *****    3. Self-report i.e. no reference to original medical records to confirm the outcome    4. No description | **1** | **Outcomes**: “The primary outcome was hospitalization with acute kidney injury … The diagnostic (ICD-10) codes used to identify the outcomes are presented in eTable 3 in the Supplement” |
| 1. *Was follow-up long enough for outcomes to occur*    1. Yes *****    2. No    3. Length of follow-up not stated | **1** | **Outcomes:** “We assessed these outcomes within 30 days of the index date (because macrolide antibiotics are prescribed for short durations and adverse events due to drug interactions would occur soon thereafter)” |
| 1. *Adequacy of follow-up of cohorts*    1. Complete follow-up – all subjects accounted for *****    2. Loss to follow-up less than 20% or description given for those lost *****    3. Follow-up rate less than 50% and no description of those lost    4. No statement | **1** | **Results:** No loss to follow up. Retrospective study. |
| **TOTAL SCORE** | **9** |  |

**MODIFIED NEWCASTLE - OTTAWA QUALITY ASSESSMENT SCALE: COHORT STUDIES**

**EndNote number:** #1149 **Author:** Gyamlani et al., 2019 **Title:** Vancomycin-associated acute kidney injury in a large veteran population

Note: A study can be awarded a maximum of one star for each numbered item within the Selection and Outcome categories. A maximum of two stars can be given for Comparability

| **Criterion and Decision rules** | **Score (Star*=1 point. Two stars**=2points. No star= 0 point)** | **Location in text** |
| --- | --- | --- |
| **SELECTION** |  |  |
| 1. *Representativeness of the exposed cohort*    1. Truly representative of the elderly population (at least 65 years old) *****    2. Subgroup of the exposed population are elderly (at least 65 years) *****    3. Exposed population was under 65 years    4. No description of the derivation of the exposed cohort | **1** | **Study setting and cohort definition**: “… data was obtained from RCAV study which examined outcomes in US veterans …”.  **Results**: Mean age was 68 years |
| 1. *Selection of the non-exposed cohort*    1. Drawn from the same community as the exposed cohort *****    2. Drawn from a different source    3. No description of the derivation of the non-exposed cohort | **1** | **Study setting and cohort definition: “**From the same cohort, we identified patients who received …” |
| 1. *Ascertainment of exposure*    1. Secure record (i.e. medical records) describing initial and/or ongoing exposure to antimicrobial *****    2. Self-reporting of exposure    3. No description of exposure | **1** | **Exposures and covariates:** “We identified vancomycin and non-glycopeptide exposures from VA pharmacy dispensation records …” |
| 1. *Demonstration of the absence of the outcome of interest at the beginning of the study*    1. Yes*    2. No | **1** | **Study setting and cohort definition:** “We included inpatients … who had no AKI events and at least one steady state trough serum vancomycin concentration within 48 hrs” |
| **COMPARABILITY** |  |  |
| 1. *Comparability of cohorts based on design and analysis*    1. Study controls for sex, age, and disease severity at baseline *    2. Study controls for the above plus any other additional factors ******    3. Limited or no attempt to control for differences between the cohorts | **2** | **Statistical analysis**: “Models were adjusted sequentially for demographic characteristics, co-morbidities, medications, blood pressure and BMI” |
| **OUTCOME** |  |  |
| 1. *Assessment of Outcome*     1. Independent or blind assessment, or confirmation of the outcome by reference to secure records (i.e. medical records, laboratory results etc) *****    2. Identified through ICD codes on database records *****    3. Self-report i.e. no reference to original medical records to confirm the outcome    4. No description | **1** | **Outcomes:** “AKI was defined by … at least 50% increase in serum creatinine from baseline and staged using KDIGO criteria from date of first antibiotic exposure.” |
| 1. *Was follow-up long enough for outcomes to occur*    1. Yes *****    2. No    3. Length of follow-up not stated | **1** | **Exposure and covariates:** “The main exposure variable was the highest recorded trough vancomycin level within 48 hrs of after initiation of therapy” |
| 1. *Adequacy of follow-up of cohorts*    1. Complete follow-up – all subjects accounted for *****    2. Loss to follow-up less than 20% or description given for those lost *****    3. Follow-up rate less than 50% and no description of those lost    4. No statement | **1** | **Results:** Retrospective study therefore no loss to follow up. |
| **TOTAL SCORE** | **9** |  |

**MODIFIED NEWCASTLE - OTTAWA QUALITY ASSESSMENT SCALE: COHORT STUDIES**

**EndNote number:** #767 **Author:** Hall et al., 2014 **Title:** Impact of empiric weight-based vancomycin dosing on nephrotoxicity and mortality in geriatric patients with methicillin resistant Staphylococcus aureus bacteraemia

Note: A study can be awarded a maximum of one star for each numbered item within the Selection and Outcome categories. A maximum of two stars can be given for Comparability

| **Criterion and Decision rules** | **Score (Star*=1 point. Two stars**=2points. No star= 0 point)** | **Location in text** |
| --- | --- | --- |
| **SELECTION** |  |  |
| 1. *Representativeness of the exposed cohort*    1. Truly representative of the elderly population (at least 65 years old) *****    2. Subgroup of the exposed population are elderly (at least 65 years) *****    3. Exposed population was under 65 years    4. No description of the derivation of the exposed cohort | **1** | **Study location and patients:** “We included all patients 1) who received vancomycin for at least 48 hours, 2) were 65 years of age or older, and 3) had MRSA bacteraemia, confirmed by microbiologic records.” |
| 1. *Selection of the non-exposed cohort*    1. Drawn from the same community as the exposed cohort *****    2. Drawn from a different source    3. No description of the derivation of the non-exposed cohort | **1** | **Study definitions:** “The comparator group was comprised of all other study eligible patients who received lower vancomycin doses.” |
| 1. *Ascertainment of exposure*    1. Secure record (i.e. medical records) describing initial and/or ongoing exposure to antimicrobial *****    2. Self-reporting of exposure    3. No description of exposure | **1** | **Study location and patients:** “We included all patients 1) who received vancomycin for at least 48 hours …” |
| 1. *Demonstration of the absence of the outcome of interest at the beginning of the study*    1. Yes *****    2. No | **1** | **Study definitions**: “Nephrotoxicity was defined as an increase in serum creatinine (SCr) by greater than 0.5 mg/dl or a 50% increase from baseline on at least two consecutive laboratory tests from the initiation to the completion of vancomycin therapy” |
| **COMPARABILITY** |  |  |
| 1. *Comparability of cohorts based on design and analysis*    1. Study controls for sex, age, and disease severity at baseline *    2. Study controls for the above plus any other additional factors ******    3. Limited or no attempt to control for differences between the cohorts | **2** | **Statistical analysis:** “Any variable demonstrating a 10% change in the exposure effect was determined to be a confounder and kept in the final model”  **Table 2:** Included for multivariable analysis were Guideline-recommended vancomycin dosing, Patient age, Pitt bacteraemia score of 4, Intensive care unit residence, Concomitant nephrotoxins” |
| **OUTCOME** |  |  |
| 1. *Assessment of Outcome*     1. Independent or blind assessment, or confirmation of the outcome by reference to secure records (i.e. medical records, laboratory results etc) *****    2. Identified through ICD codes on database records *****    3. Self-report i.e. no reference to original medical records to confirm the outcome    4. No description | **1** | **Study definitions:** “Nephrotoxicity was defined as an increase in serum creatinine (SCr) by greater than 0.5 mg/dl or a 50% increase from baseline on at least two consecutive laboratory tests from the initiation to the completion of vancomycin therapy” |
| 1. *Was follow-up long enough for outcomes to occur*    1. Yes *****    2. No    3. Length of follow-up not stated | **1** | **Statistical analysis:** 15 days or more “…vancomycin duration greater than 15 days” |
| 1. *Adequacy of follow-up of cohorts*    1. Complete follow-up – all subjects accounted for *****    2. Loss to follow-up less than 20% or description given for those lost *****    3. Follow-up rate less than 50% and no description of those lost    4. No statement | **1** | **Results:** Retrospective analysis. No loss to follow up |
| **TOTAL SCORE** | **9** |  |

**MODIFIED NEWCASTLE - OTTAWA QUALITY ASSESSMENT SCALE: COHORT STUDIES**

**EndNote number:** #717 **Author:** Huang et al., 2018 **Title:** Efficacy of Vancomycin on Gram-Positive Bacterial Infection in Elderly Critical Patients and Risk Factors Associated with Nephrotoxicity

Note: A study can be awarded a maximum of one star for each numbered item within the Selection and Outcome categories. A maximum of two stars can be given for Comparability

| **Criterion and Decision rules** | **Score (Star*=1 point. Two stars**=2points. No star= 0 point)** | **Location in text** |
| --- | --- | --- |
| **SELECTION** |  |  |
| 1. *Representativeness of the exposed cohort*    1. Truly representative of the elderly population (at least 65 years old) *****    2. Subgroup of the exposed population are elderly (at least 65 years) *****    3. Exposed population was under 65 years    4. No description of the derivation of the exposed cohort | **1** | **Materials and methods: “**Inclusion criteria were age ≥80 years, receiving vancomycin by intermittent infusion, intravenous vancomycin therapy for at least 4 doses, and the course of vancomycin treatment more than 72 hours.” |
| 1. *Selection of the non-exposed cohort*    1. Drawn from the same community as the exposed cohort *****    2. Drawn from a different source    3. No description of the derivation of the non-exposed cohort | **1** | Drawn from the same cohort as the exposed |
| 1. *Ascertainment of exposure*    1. Secure record (i.e. medical records) describing initial and/or ongoing exposure to antimicrobial *****    2. Self-reporting of exposure    3. No description of exposure | **1** | **Materials and methods: “**Inclusion criteria were age ≥80 years, receiving vancomycin by intermittent infusion, intravenous vancomycin therapy for at least 4 doses, and the course of vancomycin treatment more than 72 hours” |
| 1. *Demonstration of the absence of the outcome of interest at the beginning of the study*    1. Yes *****    2. No | **1** | **Subjects and data collection**: “The following laboratory findings before, during and after treatment were collected: haematologic properties, routine biochemical tests, C-reactive protein, and hepatic and renal function tests.” |
| **COMPARABILITY** |  |  |
| 1. *Comparability of cohorts based on design and analysis*    1. Study controls for sex, age, and disease severity at baseline *    2. Study controls for the above plus any other additional factors ******    3. Limited or no attempt to control for differences between the cohorts | **0** | **Statistical analysis: “**Univariate and multivariable logistic regression analyses were used to investigate the relationship amongst factors …” but no mention of apriori and other confounding variables controlled for. |
| **OUTCOME** |  |  |
| 1. *Assessment of Outcome*     1. Independent or blind assessment, or confirmation of the outcome by reference to secure records (i.e. medical records, laboratory results etc) *****    2. Identified through ICD codes on database records *****    3. Self-report i.e. no reference to original medical records to confirm the outcome    4. No description | **1** | **Evaluation of nephrotoxicity: “**The occurrence of nephrotoxicity was defined as an increase in Scr levels of 44.2 μmol/L or a 50% increase, whichever was greater, on at least 2 consecutive days during the period from initiation of vancomycin therapy to 72 hours after the completion of therapy…” |
| 1. *Was follow-up long enough for outcomes to occur*    1. Yes *****    2. No    3. Length of follow-up not stated | **1** | **Evaluation of nephrotoxicity: “**The occurrence of nephrotoxicity was defined as an increase in Scr levels of 44.2 μmol/L or a 50% increase, whichever was greater, on at least 2 consecutive days during the period from initiation of vancomycin therapy to 72 hours after the completion of therapy.” |
| 1. *Adequacy of follow-up of cohorts*    1. Complete follow-up – all subjects accounted for *****    2. Loss to follow-up less than 20% or description given for those lost *****    3. Follow-up rate less than 50% and no description of those lost    4. No statement | **1** | **Results:** No loss to follow up. Retrospective study. |
| **TOTAL SCORE** | **7** |  |

**MODIFIED NEWCASTLE - OTTAWA QUALITY ASSESSMENT SCALE: COHORT STUDIES**

**EndNote number:**  #495 **Author:** Karino et al., 2014 **Title:** Nephrotoxicity Induced by Piperacillin–Tazobactam in Late Elderly Japanese Patients with Nursing and Healthcare Associated Pneumonia

Note: A study can be awarded a maximum of one star for each numbered item within the Selection and Outcome categories. A maximum of two stars can be given for Comparability

| **Criterion and Decision rules** | **Score (Star*=1 point. Two stars**=2points. No star= 0 point)** | **Location in text** |
| --- | --- | --- |
| **SELECTION** |  |  |
| 1. *Representativeness of the exposed cohort*    1. Truly representative of the elderly population (at least 65 years old) *****    2. Subgroup of the exposed population are elderly (at least 65 years) *****    3. Exposed population was under 65 years    4. No description of the derivation of the exposed cohort | **1** | **Subjects and study protocol:** “The patients aged 65 years old or older, who were diagnosed with NHCAP in Shimane University Hospital, and satisfying the three items of: (1) body temperature of 37°C or more, (2) C-reactive protein (CRP) value of 1.0 mg/dL or more, and (3) clear pneumonia shadow observed upon chest X-ray or computed tomographic image within 2 d prior to commencing treatment” |
| 1. *Selection of the non-exposed cohort*    1. Drawn from the same community as the exposed cohort *****    2. Drawn from a different source    3. No description of the derivation of the non-exposed cohort | **1** | Drawn from the same cohort as the exposed group |
| 1. *Ascertainment of exposure*    1. Secure record (i.e. medical records) describing initial and/or ongoing exposure to antimicrobial *****    2. Self-reporting of exposure    3. No description of exposure | **1** | **Subjects and study protocol:** “PIPC–TAZ (4.0 g/0.5 g) was dissolved in 100 mL saline and intravenous drip infusion was carried out for an hour each session three times a day” |
| 1. *Demonstration of the absence of the outcome of interest at the beginning of the study*    1. Yes *****    2. No | **1** | **Subjects and protocol: “**The primary definition of nephrotoxicity used was at least a 100% (>2-fold) increase in creatinine clearance (CLcr)” |
| **COMPARABILITY** |  |  |
| 1. *Comparability of cohorts based on design and analysis*    1. Study controls for sex, age, and disease severity at baseline *    2. Study controls for the above plus any other additional factors ******    3. Limited or no attempt to control for differences between the cohorts | **0** | **Statistical analysis:** No evidence of controlling for confounding factors given. |
| **OUTCOME** |  |  |
| 1. *Assessment of Outcome*     1. Independent or blind assessment, or confirmation of the outcome by reference to secure records (i.e. medical records, laboratory results etc) *****    2. Identified through ICD codes on database records *****    3. Self-report i.e. no reference to original medical records to confirm the outcome    4. No description | **1** | **Subjects and study protocol: “**The primary definition of nephrotoxicity used was at least a 100% (>2-fold) increase in creatinine clearance (CLcr)” |
| 1. *Was follow-up long enough for outcomes to occur*    1. Yes *****    2. No    3. Length of follow-up not stated | **1** | **Subjects and study protocol: “**Assessment of nephrotoxicity began 24 h following the first order for PIPC–TAZ.”  **Results:** “…nephrotoxicity was observed in 4 cases (18.2%) at 4–7 d after the beginning of PIPC–TAZ administration” |
| 1. *Adequacy of follow-up of cohorts*    1. Complete follow-up – all subjects accounted for *****    2. Loss to follow-up less than 20% or description given for those lost *****    3. Follow-up rate less than 50% and no description of those lost    4. No statement | **1** | **Results:** All recruited participants accounted for. |
| **TOTAL SCORE** | **7** |  |

**MODIFIED NEWCASTLE - OTTAWA QUALITY ASSESSMENT SCALE: COHORT STUDIES**

**EndNote number:** #421 **Author:** Li et al., 2015 **Title:**  Risk of adverse events among older adults following co-prescription of clarithromycin and statins not metabolized by cytochrome P450 3A4

Note: A study can be awarded a maximum of one star for each numbered item within the Selection and Outcome categories. A maximum of two stars can be given for Comparability

| **Criterion and Decision rules** | **Score (Star*=1 point. Two stars**=2points. No star= 0 point)** | **Location in text** |
| --- | --- | --- |
| **SELECTION** |  |  |
| 1. *Representativeness of the exposed cohort*    1. Truly representative of the elderly population (at least 65 years old) *****    2. Subgroup of the exposed population are elderly (at least 65 years) *****    3. Exposed population was under 65 years    4. No description of the derivation of the exposed cohort | **1** | **Study design and setting:** “We conducted a population-based, retrospective cohort study … Data on adults 66 years of age and older between June 2002 and March 2013 were obtained and analysed …” |
| 1. *Selection of the non-exposed cohort*    1. Drawn from the same community as the exposed cohort *****    2. Drawn from a different source    3. No description of the derivation of the non-exposed cohort | **1** | Selected from the same cohort as the exposed cohort |
| 1. *Ascertainment of exposure*    1. Secure record (i.e. medical records) describing initial and/or ongoing exposure to antimicrobial *****    2. Self-reporting of exposure    3. No description of exposure | **1** | **Data sources:** “We used the database of the Ontario Drug Benefit Program to identify prescription drug use. The database contains accurate records (error rate <1%) for all outpatient prescriptions dispensed to people 65 years or older” |
| 1. *Demonstration of the absence of the outcome of interest at the beginning of the study*    1. Yes *****    2. No | **0** | Authors did not show how they determined absence of outcome of interest at recruitment of participants. |
| **COMPARABILITY** |  |  |
| 1. *Comparability of cohorts based on design and analysis*    1. Study controls for sex, age, and disease severity at baseline *    2. Study controls for the above plus any other additional factors ******    3. Limited or no attempt to control for differences between the cohorts | **2** | **Statistical analysis:** “We adjusted for 15 potential confounding variables based on clinical relevance: age, sex, year of cohort entry; baseline evidence of chronic kidney disease, stroke or transient ischemic attack, peripheral vascular disease, coronary artery disease, congestive heart failure, major cancer and diabetes; baseline use, in the 120 days before the index date, of β-blockers, calcium-channel blockers, diuretics, angiotensin-converting-enzyme inhibitors or angiotensin II receptor blockers, and nonsteroidal anti-inflammatory drugs.” |
| **OUTCOME** |  |  |
| 1. *Assessment of Outcome*     1. Independent or blind assessment, or confirmation of the outcome by reference to secure records (i.e. medical records, laboratory results etc) *****    2. Identified through ICD codes on database records *****    3. Self-report i.e. no reference to original medical records to confirm the outcome    4. No description | **1** | **Data sources: “**We used ICD-10 codes to ascertain outcomes **…”** |
| 1. *Was follow-up long enough for outcomes to occur*    1. Yes *****    2. No    3. Length of follow-up not stated | **1** | **Outcomes:** “We followed patients for 30 days after the index date to assess outcomes.” |
| 1. *Adequacy of follow-up of cohorts*    1. Complete follow-up – all subjects accounted for *****    2. Loss to follow-up less than 20% or description given for those lost *****    3. Follow-up rate less than 50% and no description of those lost    4. No statement | **1** | **Results:** No loss to follow up. Prospective study**.** |
| **TOTAL SCORE** | **8** |  |

**MODIFIED NEWCASTLE - OTTAWA QUALITY ASSESSMENT SCALE: COHORT STUDIES**

**EndNote number:** #918 **Author:** Liu et al., 2015 **Title:**  Retrospective Analysis of Vancomycin Nephrotoxicity in Elderly Chinese Patients

Note: A study can be awarded a maximum of one star for each numbered item within the Selection and Outcome categories. A maximum of two stars can be given for Comparability

| **Criterion and Decision rules** | **Score (Star*=1 point. Two stars**=2points. No star= 0 point)** | **Location in text** |
| --- | --- | --- |
| **SELECTION** |  |  |
| 1. *Representativeness of the exposed cohort*    1. Truly representative of the elderly population (at least 65 years old) *****    2. Subgroup of the exposed population are elderly (at least 65 years) *****    3. Exposed population was under 65 years    4. No description of the derivation of the exposed cohort | **1** | **Methods:** “The inclusion criteria were as follows: (1) ≥ 60 years of age; (2) had an absolute neutrophil count ≥ 1,000 cells/mm 3 ; (3) had received vancomycin for ≥ 48 h; (4) had a baseline serum creatinine (SCr) value of <2.0 mg/dl”  **Table 1**: Mean age (within 1 SD) of nephrotoxic patients was about 70 (+ or -5) and 72 (+ or -7) for non-nephrotoxic patients, to the nearest year. |
| 1. *Selection of the non-exposed cohort*    1. Drawn from the same community as the exposed cohort *****    2. Drawn from a different source    3. No description of the derivation of the non-exposed cohort | **1** | Selected from the same community as the exposed cohort**.** |
| 1. *Ascertainment of exposure*    1. Secure record (i.e. medical records) describing initial and/or ongoing exposure to antimicrobial *****    2. Self-reporting of exposure    3. No description of exposure | **1** | **Methods: “**The inclusion criteria were as follows: (1) ≥ 60 years of age; (2) had an absolute neutrophil count ≥ 1,000 cells/mm 3 ; (3) had received vancomycin for ≥ 48 hours; …” |
| 1. *Demonstration of the absence of the outcome of interest at the beginning of the study*    1. Yes *****    2. No | **1** | **Methods: “**The inclusion criteria were as follows: (1) ≥ 60 years of age; … (4) had a baseline serum creatinine (SCr) value of <2.0 mg/dl” |
| **COMPARABILITY** |  |  |
| 1. *Comparability of cohorts based on design and analysis*    1. Study controls for sex, age, and disease severity at baseline *    2. Study controls for the above plus any other additional factors ******    3. Limited or no attempt to control for differences between the cohorts | **0** | **Statistical analysis:** There is no mention of adjusting for apriori and other possible confounders**.** |
| **OUTCOME** |  |  |
| 1. *Assessment of Outcome*     1. Independent or blind assessment, or confirmation of the outcome by reference to secure records (i.e. medical records, laboratory results etc) *****    2. Identified through ICD codes on database records *****    3. Self-report i.e. no reference to original medical records to confirm the outcome    4. No description | **1** | **Methods:** “Nephrotoxicity was defined using the Acute Kidney Injury Network (AKIN) Stage 1 classification either as an increase in SCr level of 0.5 mg/dl or as a 1.5–2-fold increase in SCr from baseline, whichever was greater. Patients were considered to have developed nephrotoxicity if they were classified as AKIN Stage 1 for at least 2 consecutive days during the period from initiation of vancomycin therapy to 72 h after the therapy” |
| 1. *Was follow-up long enough for outcomes to occur*    1. Yes *****    2. No    3. Length of follow-up not stated | **1** | **Methods:** “Patients were considered to have developed nephrotoxicity if they were classified as AKIN Stage 1 for at least 2 consecutive days during the period from initiation of vancomycin therapy to 72 h after the therapy” |
| 1. *Adequacy of follow-up of cohorts*    1. Complete follow-up – all subjects accounted for *****    2. Loss to follow-up less than 20% or description given for those lost *****    3. Follow-up rate less than 50% and no description of those lost    4. No statement | **1** | **Results:** All included participants accounted for in the results |
| **TOTAL SCORE** | **7** |  |

**MODIFIED NEWCASTLE - OTTAWA QUALITY ASSESSMENT SCALE: COHORT STUDIES**

**EndNote number:** #332 **Author:** Mizokami et al., 2013. **Title:**  Pharmacodynamics of vancomycin in elderly patients aged 75 years or older with methicillin-resistant Staphylococcus aureus hospital-acquired pneumonia

Note: A study can be awarded a maximum of one star for each numbered item within the Selection and Outcome categories. A maximum of two stars can be given for Comparability

| **Criterion and Decision rules** | **Score (Star*=1 point. Two stars**=2points. No star= 0 point)** | **Location in text** |
| --- | --- | --- |
| **SELECTION** |  |  |
| 1. *Representativeness of the exposed cohort*    1. Truly representative of the elderly population (at least 65 years old) *****    2. Subgroup of the exposed population are elderly (at least 65 years) *****    3. Exposed population was under 65 years    4. No description of the derivation of the exposed cohort | **1** | **Study location and patients**: “During a 6-year period (from January 2006 through December 2012), all hospitalized patients aged 75 years or older with MRSA pneumonia microbiologically confirmed by sputum or blood cultures and treated with vancomycin therapy were identified using the clinical pharmacokinetics department computer database.” |
| 1. *Selection of the non-exposed cohort*    1. Drawn from the same community as the exposed cohort *****    2. Drawn from a different source    3. No description of the derivation of the non-exposed cohort | **1** | Drawn from the same community as the exposed cohort. |
| 1. *Ascertainment of exposure*    1. Secure record (i.e. medical records) describing initial and/or ongoing exposure to antimicrobial *****    2. Self-reporting of exposure    3. No description of exposure | **1** | **Study location and patients:** “During a 6-year period (from January 2006 through December 2012), all hospitalized patients aged 75 years or older with MRSA pneumonia … and treated with vancomycin therapy were identified using the clinical pharmacokinetics department computer database.” |
| 1. *Demonstration of the absence of the outcome of interest at the beginning of the study*    1. Yes *****    2. No | **1** | **Definitions:** “Nephrotoxicity resulting from treatment with vancomycin was defined as an increase in serum creatinine of 0.5 mg/dL or a 50% increase from pre-treatment levels” |
| **COMPARABILITY** |  |  |
| 1. *Comparability of cohorts based on design and analysis*    1. Study controls for sex, age, and disease severity at baseline *    2. Study controls for the above plus any other additional factors ******    3. Limited or no attempt to control for differences between the cohorts | **0** | **Statistical analysis:** Authors did not mention control of apriori and other possible confounders**.** |
| **OUTCOME** |  |  |
| 1. *Assessment of Outcome*     1. Independent or blind assessment, or confirmation of the outcome by reference to secure records (i.e. medical records, laboratory results etc) *****    2. Identified through ICD codes on database records *****    3. Self-report i.e. no reference to original medical records to confirm the outcome    4. No description | **1** | **Definitions:** “Nephrotoxicity resulting from treatment with vancomycin was defined as an increase in serum creatinine of 0.5 mg/dL or a 50% increase from pre-treatment levels.” |
| 1. *Was follow-up long enough for outcomes to occur*    1. Yes *****    2. No    3. Length of follow-up not stated | **1** | **Statistical analysis**: “The primary data from 28-day survivors and non-survivors were compared” |
| 1. *Adequacy of follow-up of cohorts*    1. Complete follow-up – all subjects accounted for *****    2. Loss to follow-up less than 20% or description given for those lost *****    3. Follow-up rate less than 50% and no description of those lost    4. No statement | **1** | Retrospective study. No loss to follow up |
| **TOTAL SCORE** | **7** |  |

**MODIFIED NEWCASTLE - OTTAWA QUALITY ASSESSMENT SCALE: COHORT STUDIES**

**EndNote number:** #217 **Author:** Noh et al., 2019. **Title:**  Completion rate of latent tuberculosis infection treatment in patients aged 65 years and older

Note: A study can be awarded a maximum of one star for each numbered item within the Selection and Outcome categories. A maximum of two stars can be given for Comparability

| **Criterion and Decision rules** | **Score (Star*=1 point. Two stars**=2points. No star= 0 point)** | **Location in text** |
| --- | --- | --- |
| **SELECTION** |  |  |
| 1. *Representativeness of the exposed cohort*    1. Truly representative of the elderly population (at least 65 years old) *****    2. Subgroup of the exposed population are elderly (at least 65 years) *****    3. Exposed population was under 65 years    4. No description of the derivation of the exposed cohort | **1** | **Study design and patients:** “We reviewed the electronic medical records of patients aged 65 years and older who were diagnosed with LTBI between January 2016 and December 2018” |
| 1. *Selection of the non-exposed cohort*    1. Drawn from the same community as the exposed cohort *****    2. Drawn from a different source    3. No description of the derivation of the non-exposed cohort | **1** | Drawn from the same community as the exposed cohort. |
| 1. *Ascertainment of exposure*    1. Secure record (i.e. medical records) describing initial and/or ongoing exposure to antimicrobial *****    2. Self-reporting of exposure    3. No description of exposure | **1** | **Data collection and definitions:** “Treatment completion was defined as ingestion of more than 80% of all prescribed medications without loss to follow-up: within 12 months for 9H, within 6 months for 4R and 4 months for 3HR” |
| 1. *Demonstration of the absence of the outcome of interest at the beginning of the study*    1. Yes *****    2. No | **1** | **Treatment of LTBI:** “Laboratory tests (complete blood count, aspartate transaminase (AST, ALT, and total bilirubin) were performed at every visit. Korean guideline for tuberculosis recommends that anti-TB medications should be stopped if aminotransferase levels exceeded 3 times of upper normal range with symptoms of hepatitis or exceeded 5 times of upper normal range without symptoms” |
| **COMPARABILITY** |  |  |
| 1. *Comparability of cohorts based on design and analysis*    1. Study controls for sex, age, and disease severity at baseline *    2. Study controls for the above plus any other additional factors ******    3. Limited or no attempt to control for differences between the cohorts | **2** | **Statistical analysis:** “Multivariable logistic regression analysis using the backward elimination method was performed to identify the factors associated with completion of LTBI treatment. Independent variables were selected based on their statistical significance in univariate analyses and based on being clinically applicable” |
| **OUTCOME** |  |  |
| 1. *Assessment of Outcome*     1. Independent or blind assessment, or confirmation of the outcome by reference to secure records (i.e. medical records, laboratory results etc) *****    2. Identified through ICD codes on database records *****    3. Self-report i.e. no reference to original medical records to confirm the outcome    4. No description | **1** | **Data collection and definitions:** “Hepatotoxicity was graded as follows by the National Cancer Institute's Common Terminology Criteria for Adverse Effects Version 5.0: Grade 1: alanine transaminase (ALT) 1–3 times; Grade 2: ALT 3–5 times; Grade 3: ALT 3–10 times with symptoms or 5–10 times without symptoms; and Grade 4: ALT > 10 times.” |
| 1. *Was follow-up long enough for outcomes to occur*    1. Yes *****    2. No    3. Length of follow-up not stated | **1** | **Treatment of LTBI:** “During LTBI treatment, patients were followed up for the first 2 weeks and then every 4 weeks until the end of the treatment.” |
| 1. *Adequacy of follow-up of cohorts*    1. Complete follow-up – all subjects accounted for *****    2. Loss to follow-up less than 20% or description given for those lost *****    3. Follow-up rate less than 50% and no description of those lost    4. No statement | **1** | **Results:** “There were 13 (16.9%) patients who did not complete treatment, and 10 (13.0%) patients were lost to follow-up” |
| **TOTAL SCORE** | **9** |  |

**MODIFIED NEWCASTLE - OTTAWA QUALITY ASSESSMENT SCALE: COHORT STUDIES**

**EndNote number:** #253 **Author:** Ong et al., 2016. **Title:** Aminoglycoside-associated acute kidney injury in elderly patients with and without shock

Note: A study can be awarded a maximum of one star for each numbered item within the Selection and Outcome categories. A maximum of two stars can be given for Comparability

| **Criterion and Decision rules** | **Score (Star*=1 point. Two stars**=2points. No star= 0 point)** | **Location in text** |
| --- | --- | --- |
| **SELECTION** |  |  |
| 1. *Representativeness of the exposed cohort*    1. Truly representative of the elderly population (at least 65 years old) *****    2. Subgroup of the exposed population are elderly (at least 65 years) *****    3. Exposed population was under 65 years    4. No description of the derivation of the exposed cohort | **1** | **Study design and participants:** “Inpatients aged ≥60 years who received a minimum of 2 days of systemic AG (gentamicin or amikacin) therapy for various systemic infections were included in the study”  **Table 1:** Entire cohort mean age was 74 years (8 years SD) |
| 1. *Selection of the non-exposed cohort*    1. Drawn from the same community as the exposed cohort *****    2. Drawn from a different source    3. No description of the derivation of the non-exposed cohort | **1** | Drawn from same community as exposed cohort. |
| 1. *Ascertainment of exposure*    1. Secure record (i.e. medical records) describing initial and/or ongoing exposure to antimicrobial *****    2. Self-reporting of exposure    3. No description of exposure | **1** | **Study design and participants:** “ Patients were identified from the hospital laboratory information system based on AG drug levels requested” |
| 1. *Demonstration of the absence of the outcome of interest at the beginning of the study*    1. Yes *****    2. No | **1** | **Primary and secondary outcomes:** “Primary outcome was AKI, as defined by the Kidney Disease: Improving Global Outcomes (KDIGO) proposed criteria into stages 1, 2 or 3 using the peak serum creatinine (sCr) level within the reference window following AG exposure, and admission sCr prior to exposure” |
| **COMPARABILITY** |  |  |
| 1. *Comparability of cohorts based on design and analysis*    1. Study controls for sex, age, and disease severity at baseline *    2. Study controls for the above plus any other additional factors ******    3. Limited or no attempt to control for differences between the cohorts | **2** | **Table 3:** “Covariates included: AG therapy >10 days; contrast media administration (versus none); frusemide administration (versus none); pneumonia (versus other primary sources of infection); shock (versus no shock); mechanical ventilation (versus none).” |
| **OUTCOME** |  |  |
| 1. *Assessment of Outcome*     1. Independent or blind assessment, or confirmation of the outcome by reference to secure records (i.e. medical records, laboratory results etc) *****    2. Identified through ICD codes on database records *****    3. Self-report i.e. no reference to original medical records to confirm the outcome    4. No description | **1** | **Primary and secondary outcomes**: “Primary outcome was AKI, as defined by the Kidney Disease: Improving Global Outcomes (KDIGO) proposed criteria into stages 1, 2 or 3 using the peak serum creatinine (sCr) level within the reference window following AG exposure, and admission sCr prior to exposure” |
| 1. *Was follow-up long enough for outcomes to occur*    1. Yes *****    2. No    3. Length of follow-up not stated | **1** | **Primary and secondary outcomes:** “Peak sCr levels were recorded from within 1 week after first AG drug level checked until 2 weeks post-cessation of AG therapy. Acute DsCr was measured as the difference between reference peak sCr and admission sCr levels during the index hospitalization.” |
| 1. *Adequacy of follow-up of cohorts*    1. Complete follow-up – all subjects accounted for *****    2. Loss to follow-up less than 20% or description given for those lost *****    3. Follow-up rate less than 50% and no description of those lost    4. No statement | **1** | Retrospective cohort study. No losses during follow up recorded**.** |
| **TOTAL SCORE** | **9** |  |

**MODIFIED NEWCASTLE - OTTAWA QUALITY ASSESSMENT SCALE: COHORT STUDIES**

**EndNote number**: #233 **Author:** Pan et al., 2018. **Title:** Vancomycin-induced acute kidney injury in elderly Chinese patients: a single-centre cross-sectional study

Note: A study can be awarded a maximum of one star for each numbered item within the Selection and Outcome categories. A maximum of two stars can be given for Comparability

| **Criterion and Decision rules** | **Score (Star*=1 point. Two stars**=2points. No star= 0 point)** | **Location in text** |
| --- | --- | --- |
| **SELECTION** |  |  |
| 1. *Representativeness of the exposed cohort*    1. Truly representative of the elderly population (at least 65 years old) *****    2. Subgroup of the exposed population are elderly (at least 65 years) *****    3. Exposed population was under 65 years    4. No description of the derivation of the exposed cohort | **1** | **Study design and population:** “… the inclusion criteria were: (i) ≥65 years of age; (ii) receiving four or more doses of VAN during treatment period” |
| 1. *Selection of the non-exposed cohort*    1. Drawn from the same community as the exposed cohort *****    2. Drawn from a different source    3. No description of the derivation of the non-exposed cohort | **1** | Drawn from the same community as the exposed cohort. |
| 1. *Ascertainment of exposure*    1. Secure record (i.e. medical records) describing initial and/or ongoing exposure to antimicrobial *****    2. Self-reporting of exposure    3. No description of exposure | **1** | **Study design and population:** “… the inclusion criteria were: (i) ≥65 years of age; (ii) receiving four or more doses of VAN during treatment period” |
| 1. *Demonstration of the absence of the outcome of interest at the beginning of the study*    1. Yes *****    2. No | **1** | **Study and design population:** Baseline serum creatinine measurement. “…an increase in SCr by ≥0.3 mg dl (≥26.5 μmol/l) within 48 h or an increase in SCr to ≥1.5 times baseline which is known or presumed to have occurred within the prior 7 days.” |
| **COMPARABILITY** |  |  |
| 1. *Comparability of cohorts based on design and analysis*    1. Study controls for sex, age, and disease severity at baseline *    2. Study controls for the above plus any other additional factors ******    3. Limited or no attempt to control for differences between the cohorts | **2** | **Data analysis:** “The covariates included in multiple logistic regression analysis of VI-AKI incidence included: age; baseline SCr; serum albumin valley; hyperuricaemia5; LOS (days); ICU admittance (yes or no); SOFA (yes or no); shock (yes or no), mechanical ventilation (yes or no); variety of VAN (Wen kexin or Lai kexin); TDM (yes or no); VAN dose adjustment (yes or no); and use of nitrates (yes or no), vasopressors (yes or no), β-blockers (yes or no), spironolactone (yes or no), furosemide (yes or no), carbapenems (yes or no), compound sulfamethoxazole (yes or no), metronidazole/ornidazole (yes or no), azole antifungal agents (yes or no), steroids (yes or no), glutathione (yes or no), acetylcysteine (yes or no) and phosphocreatine(yes or no)” |
| **OUTCOME** |  |  |
| 1. *Assessment of Outcome*     1. Independent or blind assessment, or confirmation of the outcome by reference to secure records (i.e. medical records, laboratory results etc) *****    2. Identified through ICD codes on database records *****    3. Self-report i.e. no reference to original medical records to confirm the outcome    4. No description | **1** | **Study design and population:** “We used the 2012 Kidney Disease: Improving Global Outcomes (KDIGO) definition of AKI as the major screening criterion: an increase in SCr by ≥0.3 mg/dl (≥26.5 μmol/l ) within 48 h or an increase in SCr to ≥1.5 times baseline which is known or presumed to have occurred within the prior 7 days” |
| 1. *Was follow-up long enough for outcomes to occur*    1. Yes *****    2. No    3. Length of follow-up not stated | **1** | **Study design and population:** “The definition of VI-AKI is the development of AKI during VAN therapy or within 3 days after the withdrawal of VAN.” |
| 1. *Adequacy of follow-up of cohorts*    1. Complete follow-up – all subjects accounted for *****    2. Loss to follow-up less than 20% or description given for those lost *****    3. Follow-up rate less than 50% and no description of those lost    4. No statement | **1** | Retrospective cohort study. No recorded loss to follow up. |
| **TOTAL SCORE** | **9** |  |

**MODIFIED NEWCASTLE - OTTAWA QUALITY ASSESSMENT SCALE: COHORT STUDIES**

**EndNote number**: #234 **Author:** Pan et al., 2017. **Title:** Vancomycin-associated acute kidney injury: A cross-sectional study from a single centre in China

Note: A study can be awarded a maximum of one star for each numbered item within the Selection and Outcome categories. A maximum of two stars can be given for Comparability

| **Criterion and Decision rules** | **Score (Star*=1 point. Two stars**=2points. No star= 0 point)** | **Location in text** |
| --- | --- | --- |
| **SELECTION** |  |  |
| 1. *Representativeness of the exposed cohort*    1. Truly representative of the elderly population (at least 65 years old) *****    2. Subgroup of the exposed population are elderly (at least 65 years) *****    3. Exposed population was under 65 years    4. No description of the derivation of the exposed cohort | **1** | **Survey design:** “. First, we screened the patients treated with VAN at our hospital; all patients > or = 18 years old were included.  **Table 1:** Age-stratified presentation of data and subgroup analysis |
| 1. *Selection of the non-exposed cohort*    1. Drawn from the same community as the exposed cohort *****    2. Drawn from a different source    3. No description of the derivation of the non-exposed cohort | **1** | Drawn from same community as exposed cohort. |
| 1. *Ascertainment of exposure*    1. Secure record (i.e. medical records) describing initial and/or ongoing exposure to antimicrobial *****    2. Self-reporting of exposure    3. No description of exposure | **1** | **Survey design:** “First, we screened the patients treated with VAN at our hospital; all patients > or = 18 years old were included.” |
| 1. *Demonstration of the absence of the outcome of interest at the beginning of the study*    1. Yes *****    2. No | **1** | **Survey design: “…**we further excluded patients who developed AKI before receiving VAN treatment or more than 7 days after stopping treatment.” |
| **COMPARABILITY** |  |  |
| 1. *Comparability of cohorts based on design and analysis*    1. Study controls for sex, age, and disease severity at baseline *    2. Study controls for the above plus any other additional factors ******    3. Limited or no attempt to control for differences between the cohorts | **2** | **Data analysis**: “The covariates included in multiple logistic regression analysis of VA-AKI incidence included sex, age, department, CHD, baseline SCr, reason for VAN therapy, LOS (days), ICU admittance, cancer, concomitant low perfusion factors, receipt of orthopaedic/trauma/burn surgery and other concomitant situations during hospitalization, LOT (days), mean daily dosage, and receipt of TDM, vasopressors, nitrates, β-receptor blockers, ACEIs, aminoglycosides azole antifungals, imipenem-cystatins, diuretics, 20% mannitol, contrast medium, compound glycyrrhizin, ursodeoxycholic acid, etc” |
| **OUTCOME** |  |  |
| 1. *Assessment of Outcome*     1. Independent or blind assessment, or confirmation of the outcome by reference to secure records (i.e. medical records, laboratory results etc) *****    2. Identified through ICD codes on database records *****    3. Self-report i.e. no reference to original medical records to confirm the outcome    4. No description | **1** | **Survey design:** “We used the 2012 Kidney Disease: Improving Global Outcomes (KDIGO) definition of AKI as the major screening criterion: increase in SCr by > or = 0.3 mg/dl (> or = 26.5 μmol/l) within 48 hours or increase in SCr to > or = 1.5 times baseline which is known or presumed to have occurred within the prior 7 days.” |
| 1. *Was follow-up long enough for outcomes to occur*    1. Yes *****    2. No    3. Length of follow-up not stated | **1** | **Survey design:** “The definition of VA-AKI was defined as the development of AKI during VAN treatment or within 7 days following the termination of VAN treatment” |
| 1. *Adequacy of follow-up of cohorts*    1. Complete follow-up – all subjects accounted for *****    2. Loss to follow-up less than 20% or description given for those lost *****    3. Follow-up rate less than 50% and no description of those lost    4. No statement | **1** | No loss to follow up recorded. Retrospective study. |
| **TOTAL SCORE** | **9** |  |

**MODIFIED NEWCASTLE - OTTAWA QUALITY ASSESSMENT SCALE: COHORT STUDIES**

**EndNote number:** #219.  **Author:** Paterson et al., 1998.  **Title:**  Risk factors for toxicity in elderly patients given aminoglycosides once daily

Note: A study can be awarded a maximum of one star for each numbered item within the Selection and Outcome categories. A maximum of two stars can be given for Comparability

| **Criterion and Decision rules** | **Score (Star*=1 point. Two stars**=2points. No star= 0 point)** | **Location in text** |
| --- | --- | --- |
| **SELECTION** |  |  |
| 1. *Representativeness of the exposed cohort*    1. Truly representative of the elderly population (at least 65 years old) *****    2. Subgroup of the exposed population are elderly (at least 65 years) *****    3. Exposed population was under 65 years    4. No description of the derivation of the exposed cohort | **1** | **Methods: “**Consecutive patients aged 70 years or over who were receiving aminoglycosides and were not in the intensive care or haemodialysis units were included in this study.” |
| 1. *Selection of the non-exposed cohort*    1. Drawn from the same community as the exposed cohort *****    2. Drawn from a different source    3. No description of the derivation of the non-exposed cohort | **1** | Drawn from same community as exposed cohort. |
| 1. *Ascertainment of exposure*    1. Secure record (i.e. medical records) describing initial and/or ongoing exposure to antimicrobial *****    2. Self-reporting of exposure    3. No description of exposure | **1** | **Methods: “**Consecutive patients aged 70 years or over who were receiving aminoglycosides and were not in the intensive care or haemodialysis units were included in this study.” |
| 1. *Demonstration of the absence of the outcome of interest at the beginning of the study*    1. Yes *****    2. No | **1** | **Methods:** “Serum creatinine levels were measured at the commencement of aminoglycosides in all patients, and creatinine clearance was determined by the use of Cockcroft-Gault formula.” |
| **COMPARABILITY** |  |  |
| 1. *Comparability of cohorts based on design and analysis*    1. Study controls for sex, age, and disease severity at baseline *    2. Study controls for the above plus any other additional factors ******    3. Limited or no attempt to control for differences between the cohorts | **2** | **Methods:** “Potential risk factors for nephrotoxicity were analysed using logistic regression …”  **Results:** “When multivariable analysis was done, utilizing age, duration of therapy, concomitant use of allopurinol, baseline creatinine level, and hypotension during the aminoglycoside course in the model …” |
| **OUTCOME** |  |  |
| 1. *Assessment of Outcome*     1. Independent or blind assessment, or confirmation of the outcome by reference to secure records (i.e. medical records, laboratory results etc) *****    2. Identified through ICD codes on database records *****    3. Self-report i.e. no reference to original medical records to confirm the outcome    4. No description | **1** | **Methods:** “Nephrotoxicity was defined as a rise in serum creatinine above the baseline level of 0.5 mg/dl (0.045 mmol/l) or more” |
| 1. *Was follow-up long enough for outcomes to occur*    1. Yes *****    2. No    3. Length of follow-up not stated | **1** | **Methods**: “Serum creatinine levels were measured every 2 days during aminoglycoside course and for 7 days after completion” |
| 1. *Adequacy of follow-up of cohorts*    1. Complete follow-up – all subjects accounted for *****    2. Loss to follow-up less than 20% or description given for those lost *****    3. Follow-up rate less than 50% and no description of those lost    4. No statement | **1** | **Results:** All subjects accounted for, therefore no loss to follow up |
| **TOTAL SCORE** | **9** |  |

**MODIFIED NEWCASTLE - OTTAWA QUALITY ASSESSMENT SCALE: COHORT STUDIES**

**EndNote number:** #652. **Author:** Raveh et al., 2002.  **Title:** Risk factors for nephrotoxicity in elderly patients receiving once-daily aminoglycosides

Note: A study can be awarded a maximum of one star for each numbered item within the Selection and Outcome categories. A maximum of two stars can be given for Comparability

| **Criterion and Decision rules** | **Score (Star*=1 point. Two stars**=2points. No star= 0 point)** | **Location in text** |
| --- | --- | --- |
| **SELECTION** |  |  |
| 1. *Representativeness of the exposed cohort* 2. Truly representative of the elderly population (at least 65 years old) ***** 3. Subgroup of the exposed population are elderly (at least 65 years) ***** 4. Exposed population was under 65 years 5. No description of the derivation of the exposed cohort | **1** | **Methods:** “In this prospective, non-interventional study, we screened all in- patients receiving aminoglycoside treatment (gentamicin and amikacin)”  **Table 2:** Results presented in age subgroups i.e. under 65 years and equal to or more than 65 years |
| 1. *Selection of the non-exposed cohort*    1. Drawn from the same community as the exposed cohort *****    2. Drawn from a different source    3. No description of the derivation of the non-exposed cohort | **1** | Drawn from same community as exposed cohort. |
| 1. *Ascertainment of exposure*    1. Secure record (i.e. medical records) describing initial and/or ongoing exposure to antimicrobial *****    2. Self-reporting of exposure    3. No description of exposure | **1** | **Methods: “…**we screened all in- patients receiving aminoglycoside treatment (gentamicin and amikacin)” |
| 1. *Demonstration of the absence of the outcome of interest at the beginning of the study*    1. Yes *****    2. No | **1** | **Methods:** “The parameters recorded in the questionnaire were … and creatinine clearance …” |
| **COMPARABILITY** |  |  |
| 1. *Comparability of cohorts based on design and analysis*    1. Study controls for sex, age, and disease severity at baseline *    2. Study controls for the above plus any other additional factors ******    3. Limited or no attempt to control for differences between the cohorts | **1** | **Methods**: “Parameters recorded included patient age, gender, BMI, and creatinine clearance estimated by clinicians” |
| **OUTCOME** |  |  |
| 1. *Assessment of Outcome*     1. Independent or blind assessment, or confirmation of the outcome by reference to secure records (i.e. medical records, laboratory results etc) *****    2. Identified through ICD codes on database records *****    3. Self-report i.e. no reference to original medical records to confirm the outcome    4. No description | **1** | **Methods**: “A significant deterioration in renal function was defined as an increase in serum creatinine of more than 50% from prior to treatment to maximal post initiation of treatment creatinine levels.” |
| 1. *Was follow-up long enough for outcomes to occur*    1. Yes *****    2. No    3. Length of follow-up not stated | **0** | Length of follow up not clearly stated. |
| 1. *Adequacy of follow-up of cohorts*    1. Complete follow-up – all subjects accounted for *****    2. Loss to follow-up less than 20% or description given for those lost *****    3. Follow-up rate less than 50% and no description of those lost    4. No statement | **1** | All included subjects accounted for by the authors. |
| **TOTAL SCORE** | **6** |  |

**MODIFIED NEWCASTLE - OTTAWA QUALITY ASSESSMENT SCALE: COHORT STUDIES_TEMPLATE**

**EndNote number: #a Author: Aggrawal, 2018. Title: Comparison of nephrotoxicity of colistin with polymyxin B administered in currently recommended doses: a prospective study.**

**Note:** A study can be awarded a maximum of one star for each numbered item within the Selection and Outcome categories. A maximum of two stars can be given for Comparability

| **Criterion and Decision rules** | **Score (Star*=1 point. Two stars**=2points. No star= 0 point)** | **Location in text** |
| --- | --- | --- |
| **SELECTION** |  |  |
| 1. *Representativeness of the exposed cohort*    1. Truly representative of the elderly population (at least 65 years old) *****    2. Subgroup of the exposed population are elderly (at least 65 years) *****    3. Exposed population was under 65 years    4. No description of the derivation of the exposed cohort | **0** | Hospital based study |
| 1. *Selection of the non-exposed cohort*    1. Drawn from the same community as the exposed cohort *****    2. Drawn from a different source    3. No description of the derivation of the non-exposed cohort | **1** | **Methods:** All adult patients who received colistin or polymyxin B were included |
| 1. *Ascertainment of exposure*    1. Secure record (i.e. medical records) describing initial and/or ongoing exposure to antimicrobial *****    2. Self-reporting of exposure    3. No description of exposure | **1** | **Methods:** Prospective study … all adults who received colistin or polymyxin B |
| 1. *Demonstration of the absence of the outcome of interest at the beginning of the study*    1. Yes *****    2. No | **1** | **Methods:** Excluded patients already on nephrotoxic drugs, fluctuating renal parameters, measure baseline parameters |
| **COMPARABILITY** |  |  |
| 1. *Comparability of cohorts based on design and analysis*    1. Study controls for sex, age, and disease severity at baseline *****    2. Study controls for the above plus any other additional factors ******    3. Limited or no attempt to control for differences between the cohorts | **2** | **Methods:** Confounding variables controlled for were ‘age, gender, BMI, APACHE 2, diabetes melitus, and antibiotic dose |
| **OUTCOME** |  |  |
| 1. *Assessment of Outcome*     1. Independent or blind assessment, or confirmation of the outcome by reference to secure records (i.e. medical records, laboratory results etc) *****    2. Identified through ICD codes on database records *****    3. Self-report i.e. no reference to original medical records to confirm the outcome    4. No description | **1** | **Methods:** Nephrotoxicity/renal failure defined as two fold increase in serum creatinine or 50% drop in creatinine clearance. |
| 1. *Was follow-up long enough for outcomes to occur*    1. Yes *****    2. No    3. Length of follow-up not stated | **1** | **Methods:** Serum creatinine measured for a minimum of 7 days following drug exposure. |
| 1. *Adequacy of follow-up of cohorts*    1. Complete follow-up – all subjects accounted for *****    2. Loss to follow-up less than 20% or description given for those lost *****    3. Follow-up rate less than 50% and no description of those lost    4. No statement | **1** | **Results:** Same number of participants that met the inclusion criteria were reported on. |
| **TOTAL SCORE** | **8** |  |

**MODIFIED NEWCASTLE - OTTAWA QUALITY ASSESSMENT SCALE: COHORT STUDIES_TEMPLATE**

**EndNote number: #b Author: Morimoto, 2017. Title: Frequency of acute kidney injury caused by tazobactam/piperacillin in patients with pneumonia and chronic kidney disease: A retrospective observational study**

**Note:** A study can be awarded a maximum of one star for each numbered item within the Selection and Outcome categories. A maximum of two stars can be given for Comparability

| **Criterion and Decision rules** | **Score (Star*=1 point. Two stars**=2points. No star= 0 point)** | **Location in text** |
| --- | --- | --- |
| **SELECTION** |  |  |
| 1. *Representativeness of the exposed cohort*    1. Truly representative of the elderly population (at least 65 years old) *****    2. Subgroup of the exposed population are elderly (at least 65 years) *****    3. Exposed population was under 65 years    4. No description of the derivation of the exposed cohort | **0** | Hospital based |
| 1. *Selection of the non-exposed cohort*    1. Drawn from the same community as the exposed cohort *****    2. Drawn from a different source    3. No description of the derivation of the non-exposed cohort | **1** | **Methods:** Retrospectively recruited from the same population |
| 1. *Ascertainment of exposure*    1. Secure record (i.e. medical records) describing initial and/or ongoing exposure to antimicrobial *****    2. Self-reporting of exposure    3. No description of exposure | **1** | **Methods:** ‘Identified hospitalised patients with pneumonia and treated with piperacillin/tazobactam’. |
| 1. *Demonstration of the absence of the outcome of interest at the beginning of the study*    1. Yes *****    2. No | **0** | Not clearly stating how baseline parameters were set to rule-out absence of outcome at beginning of study |
| **COMPARABILITY** |  |  |
| 1. *Comparability of cohorts based on design and analysis*    1. Study controls for sex, age, and disease severity at baseline *    2. Study controls for the above plus any other additional factors ******    3. Limited or no attempt to control for differences between the cohorts | **2** | **Methods:** Controlled for severity of pneumonia, age, gender, BMI, concomitant antibiotics. |
| **OUTCOME** |  |  |
| 1. *Assessment of Outcome*     1. Independent or blind assessment, or confirmation of the outcome by reference to secure records (i.e. medical records, laboratory results etc) *****    2. Identified through ICD codes on database records *****    3. Self-report i.e. no reference to original medical records to confirm the outcome    4. No description | **1** | **Methods:** ‘SCr of greater than 0.3 mg/dL or 1.5-fold serum creatinine levels within 7 days’ |
| 1. *Was follow-up long enough for outcomes to occur*    1. Yes *****    2. No    3. Length of follow-up not stated | **0** | Follow time not clearly stated in the methods. |
| 1. *Adequacy of follow-up of cohorts*    1. Complete follow-up – all subjects accounted for *****    2. Loss to follow-up less than 20% or description given for those lost *****    3. Follow-up rate less than 50% and no description of those lost    4. No statement | **1** | Participants recruited retrospectively therefore no loss to follow up. |
| **TOTAL SCORE** | **6** |  |

**MODIFIED NEWCASTLE - OTTAWA QUALITY ASSESSMENT SCALE: COHORT STUDIES_TEMPLATE**

**EndNote number: #c Author: Baghaei, 2010 Title: Incidence, Clinical and epidemiological risk factors and outcome of drug-induced hepatitis due to antituberculosis agents in new cases**

**Note:** A study can be awarded a maximum of one star for each numbered item within the Selection and Outcome categories. A maximum of two stars can be given for Comparability

| **Criterion and Decision rules** | **Score (Star*=1 point. Two stars**=2points. No star= 0 point)** | **Location in text** |
| --- | --- | --- |
| **SELECTION** |  |  |
| 1. *Representativeness of the exposed cohort*    1. Truly representative of the elderly population (at least 65 years old) *****    2. Subgroup of the exposed population are elderly (at least 65 years) *****    3. Exposed population was under 65 years    4. No description of the derivation of the exposed cohort | **0** | Hospital based |
| 1. *Selection of the non-exposed cohort*    1. Drawn from the same community as the exposed cohort *****    2. Drawn from a different source    3. No description of the derivation of the non-exposed cohort | **1** | **Methods:** Participants recruited from the same hospital |
| 1. *Ascertainment of exposure*    1. Secure record (i.e. medical records) describing initial and/or ongoing exposure to antimicrobial *****    2. Self-reporting of exposure    3. No description of exposure | **1** | **Methods:** Prospectively followed up ‘Initial date of treatment recorded …’ |
| 1. *Demonstration of the absence of the outcome of interest at the beginning of the study*    1. Yes *    2. No | **1** | **Methods:** ‘Baseline LFTs were performed for all patients …’ |
| **COMPARABILITY** |  |  |
| 1. *Comparability of cohorts based on design and analysis*    1. Study controls for sex, age, and disease severity at baseline *    2. Study controls for the above plus any other additional factors ******    3. Limited or no attempt to control for differences between the cohorts | **1** | **Methods:** Controlled for age, sex, nationality, smoking history, concomitant hepatotoxic drugs, BUT not severity of tuberculosis (condition being treated) |
| **OUTCOME** |  |  |
| 1. *Assessment of Outcome*     1. Independent or blind assessment, or confirmation of the outcome by reference to secure records (i.e. medical records, laboratory results etc) *****    2. Identified through ICD codes on database records *****    3. Self-report i.e. no reference to original medical records to confirm the outcome    4. No description | **1** | **Methods:** Drug induced hepatotoxicity was confirmed by laboratory values of liver function tests. |
| 1. *Was follow-up long enough for outcomes to occur*    1. Yes *****    2. No    3. Length of follow-up not stated | **0** | **Methods:** ‘Patients were visited and monitored for hepatitis …’ BUT period of follow up not clearly stated. |
| 1. *Adequacy of follow-up of cohorts*    1. Complete follow-up – all subjects accounted for *****    2. Loss to follow-up less than 20% or description given for those lost *****    3. Follow-up rate less than 50% and no description of those lost    4. No statement | **1** | **Results:** No loss to follow up recorded and all patients accounted for. |
| **TOTAL SCORE** | **6** |  |

**MODIFIED NEWCASTLE - OTTAWA QUALITY ASSESSMENT SCALE: COHORT STUDIES_TEMPLATE**

**EndNote number: #d Author: Giri, 2016 Title: Risk of acute kidney injury with amikacin versus gentamycin both in combination with metronidazolefor surgical prophylaxis**

**Note:** A study can be awarded a maximum of one star for each numbered item within the Selection and Outcome categories. A maximum of two stars can be given for Comparability

| **Criterion and Decision rules** | **Score (Star*=1 point. Two stars**=2points. No star= 0 point)** | **Location in text** |
| --- | --- | --- |
| **SELECTION** |  |  |
| 1. *Representativeness of the exposed cohort*    1. Truly representative of the elderly population (at least 65 years old) *****    2. Subgroup of the exposed population are elderly (at least 65 years) *****    3. Exposed population was under 65 years    4. No description of the derivation of the exposed cohort | **0** | **Methods**: Hospital based |
| 1. *Selection of the non-exposed cohort*    1. Drawn from the same community as the exposed cohort *****    2. Drawn from a different source    3. No description of the derivation of the non-exposed cohort | **1** | **Methods:** Drawn from the same population |
| 1. *Ascertainment of exposure*    1. Secure record (i.e., medical records) describing initial and/or ongoing exposure to antimicrobial *****    2. Self-reporting of exposure    3. No description of exposure | **1** | **Methods:** Patients prospectively given amikacin/gentamicin with metronidazole prophylaxis. |
| 1. *Demonstration of the absence of the outcome of interest at the beginning of the study*    1. Yes *****    2. No | **1** | **Methods:** ‘Before surgery baseline serum creatinine level was estimated.’ |
| **COMPARABILITY** |  |  |
| 1. *Comparability of cohorts based on design and analysis*    1. Study controls for sex, age, and disease severity at baseline *    2. Study controls for the above plus any other additional factors ******    3. Limited or no attempt to control for differences between the cohorts | **0** | Control for differences between cohorts not clearly stated. |
| **OUTCOME** |  |  |
| 1. *Assessment of Outcome*     1. Independent or blind assessment, or confirmation of the outcome by reference to secure records (i.e. medical records, laboratory results etc) *****    2. Identified through ICD codes on database records *****    3. Self-report i.e. no reference to original medical records to confirm the outcome    4. No description | **1** | **Methods:** ‘AKI was defined as sudden decrease in renal function ie rise in serum creatinine by at least 50% within 48 hours’ |
| 1. *Was follow-up long enough for outcomes to occur*    1. Yes *****    2. No    3. Length of follow-up not stated | **1** | **Methods: ‘**Follow up was done at one month ...’ |
| 1. *Adequacy of follow-up of cohorts*    1. Complete follow-up – all subjects accounted for *****    2. Loss to follow-up less than 20% or description given for those lost *****    3. Follow-up rate less than 50% and no description of those lost    4. No statement | **1** | **Results:** All subjects accounted for (table 2). |
| **TOTAL SCORE** | **6** |  |

**MODIFIED NEWCASTLE - OTTAWA QUALITY ASSESSMENT SCALE: COHORT STUDIES_TEMPLATE**

**EndNote number: #e Author: Sia, 2018 Title: Renal safety of short-term empiric gentamicin therapy in aged patients**

**Note:** A study can be awarded a maximum of one star for each numbered item within the Selection and Outcome categories. A maximum of two stars can be given for Comparability

| **Criterion and Decision rules** | **Score (Star*=1 point. Two stars**=2points. No star= 0 point)** | **Location in text** |
| --- | --- | --- |
| **SELECTION** |  |  |
| 1. *Representativeness of the exposed cohort*    1. Truly representative of the elderly population (at least 65 years old) *****    2. Subgroup of the exposed population are elderly (at least 65 years) *****    3. Exposed population was under 65 years    4. No description of the derivation of the exposed cohort | **0** | Hospital based study |
| 1. *Selection of the non-exposed cohort*    1. Drawn from the same community as the exposed cohort *****    2. Drawn from a different source    3. No description of the derivation of the non-exposed cohort | **1** | **Methods:** Drawn from the same population as the exposed cohort. |
| 1. *Ascertainment of exposure*    1. Secure record (i.e. medical records) describing initial and/or ongoing exposure to antimicrobial *****    2. Self-reporting of exposure    3. No description of exposure | **1** | **Methods:** Using the electronic dispensary system, Pyxis Medstation, to ascertain first dose of gentamicin. |
| 1. *Demonstration of the absence of the outcome of interest at the beginning of the study*    1. Yes *****    2. No | **1** | **Methods:** ‘… admission serum creatinine and eGFR at first dose of gentamicin were used as baseline.**’** |
| **COMPARABILITY** |  |  |
| 1. *Comparability of cohorts based on design and analysis*    1. Study controls for sex, age, and disease severity at baseline *    2. Study controls for the above plus any other additional factors ******    3. Limited or no attempt to control for differences between the cohorts | **2** | **Results:** Controlled for age, gender, disease severity, indication for treatment, others**.** |
| **OUTCOME** |  |  |
| *6) Assessment of Outcome*   1. Independent or blind assessment, or confirmation of the outcome by reference to secure records (i.e. medical records, laboratory results etc) ***** 2. Identified through ICD codes on database records ***** 3. Self-report i.e. no reference to original medical records to confirm the outcome 4. No description | **1** | **Methods:** ‘Acute kidney injury defined as an increase in serum creatinine by at least 50%’ |
| *7) Was follow-up long enough for outcomes to occur*   1. Yes ***** 2. No 3. Length of follow-up not stated | **1** | **Methods:** Up to 14 days after first gentamicin dose. |
| *8) Adequacy of follow-up of cohorts*   1. Complete follow-up – all subjects accounted for ***** 2. Loss to follow-up less than 20% or description given for those lost ***** 3. Follow-up rate less than 50% and no description of those lost 4. No statement | **1** | **Results:** No loss to follow up - retrospective study. |
| **TOTAL SCORE** | **8** |  |

**MODIFIED NEWCASTLE - OTTAWA QUALITY ASSESSMENT SCALE: COHORT STUDIES_TEMPLATE**

**EndNote number: #f Author: Baik, 2020 Title: Association between tendon ruptures and use of fluoroquinolone, and other antibiotics: a 10-year retrospective study of 1 million US senior Medicare beneficiaries.**

**Note:** A study can be awarded a maximum of one star for each numbered item within the Selection and Outcome categories. A maximum of two stars can be given for Comparability

| **Criterion and Decision rules** | **Score (Star*=1 point. Two stars**=2points. No star= 0 point)** | **Location in text** |
| --- | --- | --- |
| **SELECTION** |  |  |
| 1. *Representativeness of the exposed cohort*    1. Truly representative of the elderly population (at least 65 years old) *****    2. Subgroup of the exposed population are elderly (at least 65 years) *****    3. Exposed population was under 65 years    4. No description of the derivation of the exposed cohort | **1** | **Methods:** Study participants was a random 20% sample of Medicare prescription drug coverage enrolees under old age. |
| 1. *Selection of the non-exposed cohort*    1. Drawn from the same community as the exposed cohort *****    2. Drawn from a different source    3. No description of the derivation of the non-exposed cohort | **1** | Drawn from the same source as exposed participants. |
| 1. *Ascertainment of exposure*    1. Secure record (i.e. medical records) describing initial and/or ongoing exposure to antimicrobial *****    2. Self-reporting of exposure    3. No description of exposure | **1** | **Methods:** Using the claims data as evidence of exposure to study antibiotics. |
| 1. *Demonstration of the absence of the outcome of interest at the beginning of the study*    1. Yes *****    2. No | **1** | **Methods: ‘**To obtain a cohort of patients with new tendon rupture, we excluded individuals with tendon rupture reported in the first year of their Medicare entitlement.’ |
| **COMPARABILITY** |  |  |
| 1. *Comparability of cohorts based on design and analysis*    1. Study controls for sex, age, and disease severity at baseline *    2. Study controls for the above plus any other additional factors ******    3. Limited or no attempt to control for differences between the cohorts | **1** | **Methods:** Study controlled for age, gender, rural residency, income status, etc but no disease severity. |
| **OUTCOME** |  |  |
| 1. *Assessment of Outcome*     1. Independent or blind assessment, or confirmation of the outcome by reference to secure records (i.e. medical records, laboratory results etc) *****    2. Identified through ICD codes on database records *****    3. Self-report i.e. no reference to original medical records to confirm the outcome    4. No description | **1** | **Methods**: ‘We identified patients with tendon rupture based on ICD-9-CM and ICD-10-cm codes.’ |
| 1. *Was follow-up long enough for outcomes to occur*    1. Yes *****    2. No    3. Length of follow-up not stated | **1** | **Methods:** 10 year follow up. |
| 1. *Adequacy of follow-up of cohorts*    1. Complete follow-up – all subjects accounted for *****    2. Loss to follow-up less than 20% or description given for those lost *****    3. Follow-up rate less than 50% and no description of those lost    4. No statement | **1** | **Results**: No loss to follow up recorded. Retrospective study. |
| **TOTAL SCORE** | **8** |  |

**MODIFIED NEWCASTLE - OTTAWA QUALITY ASSESSMENT SCALE: COHORT STUDIES_TEMPLATE**

**EndNote number: Author: Title: ….**

**Note:** A study can be awarded a maximum of one star for each numbered item within the Selection and Outcome categories. A maximum of two stars can be given for Comparability

| **Criterion and Decision rules** | **Score (Star*=1 point. Two stars**=2points. No star= 0 point)** | **Location in text** |
| --- | --- | --- |
| **SELECTION** |  |  |
| 1. *Representativeness of the exposed cohort*    1. Truly representative of the elderly population (at least 65 years old) *****    2. Subgroup of the exposed population are elderly (at least 65 years) *****    3. Exposed population was under 65 years    4. No description of the derivation of the exposed cohort |  |  |
| 1. *Selection of the non-exposed cohort*    1. Drawn from the same community as the exposed cohort *****    2. Drawn from a different source    3. No description of the derivation of the non-exposed cohort |  |  |
| 1. *Ascertainment of exposure*    1. Secure record (i.e. medical records) describing initial and/or ongoing exposure to antimicrobial *****    2. Self-reporting of exposure    3. No description of exposure |  |  |
| 1. *Demonstration of the absence of the outcome of interest at the beginning of the study*    1. Yes *****    2. No |  |  |
| **COMPARABILITY** |  |  |
| 1. *Comparability of cohorts based on design and analysis*    1. Study controls for sex, age, and disease severity at baseline *    2. Study controls for the above plus any other additional factors ******    3. Limited or no attempt to control for differences between the cohorts |  |  |
| **OUTCOME** |  |  |
| 1. *Assessment of Outcome*     1. Independent or blind assessment, or confirmation of the outcome by reference to secure records (i.e. medical records, laboratory results etc) *****    2. Identified through ICD codes on database records *****    3. Self-report i.e. no reference to original medical records to confirm the outcome    4. No description |  |  |
| 1. *Was follow-up long enough for outcomes to occur*    1. Yes *****    2. No    3. Length of follow-up not stated |  |  |
| 1. *Adequacy of follow-up of cohorts*    1. Complete follow-up – all subjects accounted for *****    2. Loss to follow-up less than 20% or description given for those lost *****    3. Follow-up rate less than 50% and no description of those lost    4. No statement |  |  |
| **TOTAL SCORE** |  |  |
