# Supplementary material for: Risk of antimicrobial-associated organ injury among the older adults: a systematic review and meta-analysis
Source: BMC Geriatr. 2021 Nov 1;21:617. doi: 10.1186/s12877-021-02512-3 (PMC8561875; doi:10.1186/s12877-021-02512-3)
Supplement: Supplementary file 4 — Additional file 4: Appendix 1. Modified Newcastle-Ottawa Scale: Cohort Studies tool. [file 12877_2021_2512_MOESM4_ESM.docx]

**Additional file 4_doc1_Appendices**

***Appendix 1: Modified Newcastle-Ottawa Scale: Cohort Studies tool***

**Selection**

1. *Representativeness of the exposed cohort*
2. Truly representative of the elderly population (at least 65 years old) *****
3. Subgroup of the exposed population are elderly (at least 65 years) *****
4. Exposed population was under 65 years.
5. Exposed participants drawn from non-representative group e.g., hospital patients.
6. No description of the derivation of the exposed cohort

1. *Selection of the non-exposed cohort*
2. Drawn from the same community as the exposed cohort *****
3. Drawn from a different source.
4. No description of the derivation of the non-exposed cohort

1. *Ascertainment of exposure*
2. Secure record (i.e., medical records) describing initial and/or ongoing exposure to antimicrobial *****
3. Self-reporting of exposure
4. No description of exposure

1. *Demonstration of the absence of the outcome of interest at the beginning of the study*
2. Yes *****
3. No

**Comparability**

1. *Comparability of cohorts based on design and analysis.*
2. Study controls for sex, age, and disease severity at baseline *
3. Study controls for the above plus any other additional factors ******
4. Limited or no attempt to control for differences between the cohorts.

**Outcome**

1. *Assessment of Outcome*
2. Independent or blind assessment, or confirmation of the outcome by reference to secure records (i.e. medical records, laboratory results etc) *****
3. Identified through ICD codes on database records*****
4. Self-report i.e. no reference to original medical records to confirm the outcome.
5. No description

1. *Was follow-up long enough for outcomes to occur.*
2. Yes *****
3. No
4. Length of follow-up not stated.
5. *Adequacy of follow-up of cohorts*
6. Complete follow-up – all subjects accounted for *****
7. Loss to follow-up less than 20% or description given for those lost *****
8. Follow-up rate less than 50% and no description of those lost
9. No statement

**Thresholds for converting the Newcastle-Ottawa scales to risk of bias/or quality of study.**

| **Risk/Quality** | **Threshold** |
| --- | --- |
| Low risk/Good quality | 3 or 4 stars in selection domain AND 1 or 2 stars in comparability domain AND 2 or 3 stars in outcome domain |
| Medium risk/Fair quality | 2 stars in selection domain AND 1 or 2 stars in comparability domain AND 2 or 3 stars in outcome domain |
| High risk/Poor quality | 0 or 1 star in selection domain OR 0 stars in comparability domain OR 0 or 1 stars in outcome domain |
