# Supplementary material for: Risk of antimicrobial-associated organ injury among the older adults: a systematic review and meta-analysis
Source: BMC Geriatr. 2021 Nov 1;21:617. doi: 10.1186/s12877-021-02512-3 (PMC8561875; doi:10.1186/s12877-021-02512-3)
Supplement: Supplementary file 5 — Additional file 5. Funnel plots and leave-one-meta-analysis figures. [file 12877_2021_2512_MOESM5_ESM.docx]

**Additional file 5_doc1_extrafigures**

***Funnel plots and leave-one-meta-analysis figures***


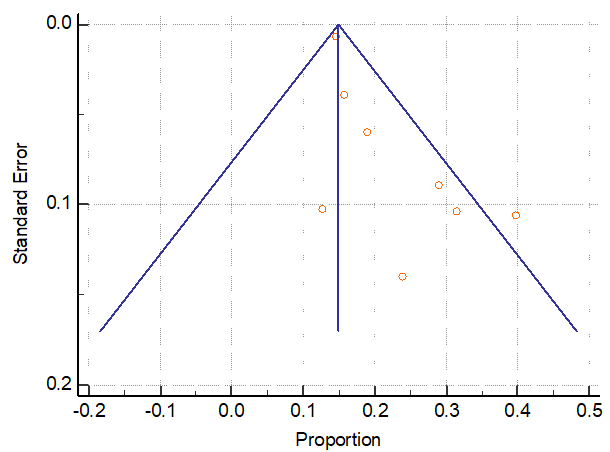


**Figure 4:** Funnel plot for studies included in the meta-analysis of the proportion of acute kidney injury among older adults prescribed glycopeptides before sensitivity analysis.


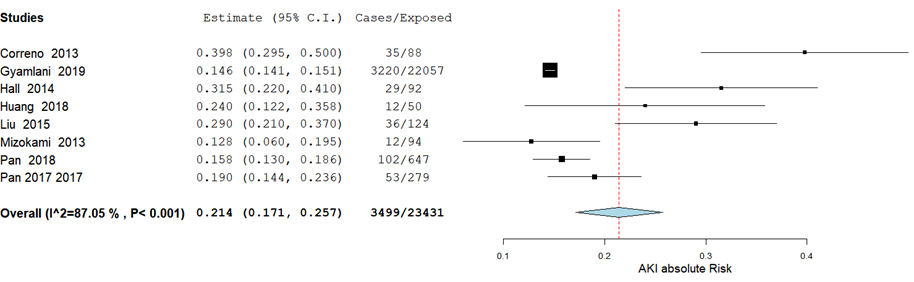


**Figure 5**: Meta-analysis of the proportion of acute kidney injury among older adults prescribed glycopeptides before sensitivity analysis


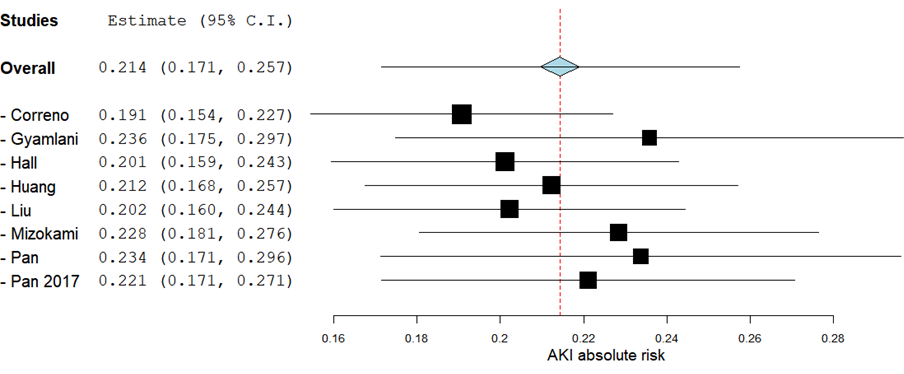


**Figure 6**: Sensitivity analysis for the studies included in the metal analysis of AKI due to glycopeptides (Leave one out approach)


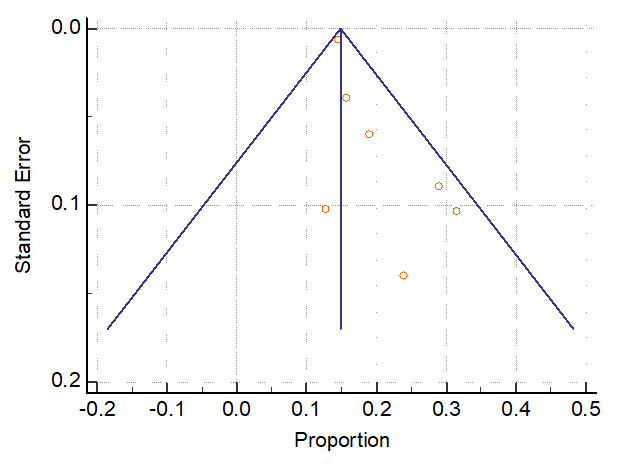


**Figure 7**: Funnel plot for studies included in the meta-analysis of the proportion of acute kidney injury among older adults prescribed glycopeptides after removing the outlier.
